# LDL receptor-peptide conjugate as in vivo tool for specific targeting of pancreatic ductal adenocarcinoma

**DOI:** 10.1038/s42003-021-02508-0

**Published:** 2021-08-19

**Authors:** Angélina Acier, Magali Godard, Fanny Gassiot, Pascal Finetti, Marion Rubis, Jonathan Nowak, François Bertucci, Juan L. Iovanna, Richard Tomasini, Pascaline Lécorché, Guillaume Jacquot, Michel Khrestchatisky, Jamal Temsamani, Cédric Malicet, Sophie Vasseur, Fabienne Guillaumond

**Affiliations:** 1 CRCM, Aix-Marseille Univ, CNRS, INSERM, Institut Paoli-Calmettes (IPC), Marseille, France; 2 Vect-Horus, Marseille, France; 3 Aix-Marseille Univ, CNRS, INP, Inst Neurophysiopathol, Marseille, France; 4 Present Address: CRCM U1068 – Pancreatic Cancer Team, 163 avenue de Luminy, Parc Scientifique de Luminy, Marseille, France

**Keywords:** Cancer metabolism, Cancer imaging

## Abstract

Despite clinical advances in diagnosis and treatment, pancreatic ductal adenocarcinoma (PDAC) remains the third leading cause of cancer death, and is still associated with poor prognosis and dismal survival rates. Identifying novel PDAC-targeted tools to tackle these unmet clinical needs is thus an urgent requirement. Here we use a peptide conjugate that specifically targets PDAC through low-density lipoprotein receptor (LDLR). We demonstrate by using near-infrared fluorescence imaging the potential of this conjugate to specifically detect and discriminate primary PDAC from healthy organs including pancreas and from benign mass-forming chronic pancreatitis, as well as detect metastatic pancreatic cancer cells in healthy liver. This work paves the way towards clinical applications in which safe LDLR-targeting peptide conjugate promotes tumor-specific delivery of imaging and/or therapeutic agents, thereby leading to substantial improvements of the PDAC patient’s outcome.

## Abstract (web summary)

Acier et al. investigated a peptide cargo system, the Fc(A680)-VH4127, that targets PDAC through the LDLR cell-surface receptor. The Fc(A680)-VH4127 was found to specifically target spontaneous pancreatic tumors in KICmice, as well as metastatic pancreatic tumors in liver.

## Introduction

Pancreatic ductal adenocarcinoma (PDAC) is a highly devastating disease with a poor 5-year overall survival rate of all stages (7–10%), and an expected rising incidence in industrialized countries^1,2^. This dismal prognosis is mainly caused by the lack of early symptoms, reliable diagnostic tools, and effective therapies, as well as early metastatic dissemination^3^. Surgical resection is the only curative treatment for patients with localized PDAC, representing 20% of all diagnosed patients. All other patients with advanced or metastatic PDAC are treated with the multi-drug regimen FOLFIRINOX or Gemcitabine/Nab-paclitaxel, both showing limited clinical efficacy and severe side effects^4^. In addition to the treatment limitations, the diagnostic performances of current imaging modalities in evaluating PDAC resectability, such as computed tomography (CT) or endoscopic ultrasonography for local staging and magnetic resonance imaging (MRI) for metastatic spread, are fairly poor at detecting small primary and metastatic tumor mass, or at distinguishing mass-forming chronic pancreatitis (CP) from poorly differentiated PDAC^5,6^. Furthermore, functional imaging with positron emission tomography (PET) using the ^18^F-fluorodeoxyglucose (FDG) radiotracer combined to computed tomography is also ineffective for acute and CP diagnosis, given that FDG is captured to the same extent by mass-forming pancreatitis and by PDAC^7,8^. Hence, both conventional treatments and diagnostic imaging modalities lack tumor-targeting specificity and consequently are poorly effective. To achieve the required specificity, the development of reliable probes and drug delivery systems that specifically target PDAC, through cell-surface receptors, is essential^9^.

In a previous study aimed to decrypt the main metabolic adaptations contributing to PDAC aggressiveness, we identified the low-density lipoprotein receptor (*Ldlr*) as one of the most upregulated metabolic transcripts involved in lipid-specific reprogramming of PDAC^10^. In genetically engineered KIC mice (*LSL-Kras*^*G12D*^; *Ink4a/Arf*^*fl/fl*^; *Pdx1-Cre*)^11^, we showed that LDLR, highly expressed at the cell-surface of PDAC cells, is the major gateway through which the tumor satisfies its cholesterol needs via an increase in cholesterol-carrying LDL uptake^10^.

Beyond its crucial role in supplying cholesterol, we investigated whether the cell-surface LDLR, which we find also highly expressed in patient PDAC and metastases, may constitute a promising target candidate to ensure specific tumor-targeting. To this end, we used a peptide-based delivery strategy, that has already proven itself in the early diagnosis, guided-surgery, and targeted therapy of PDAC^12–14^. Here, we utilized a fluorescent LDLR-targeting peptide conjugated to a human IgG Fc fragment (i.e., Fc(A680)-VH4127), already validated in engineered cells overexpressing LDLR^15^. We first demonstrate that Fc(A680)-VH4127 retains efficient LDLR-dependent endocytosis in PDAC derived-cancer cells. We also show that this conjugate specifically targets PDAC in vivo, through LDLR, without causing hepatic and renal damages, and enables the discrimination of advanced PDAC from CP and the detection of liver-disseminated pancreatic cancer cells. This work represents a proof-of-concept regarding the use of LDLR-targeting peptides as efficient tools for the specific targeting of primary and metastatic PDAC and offers new perspectives for their use in other clinical applications. After labeling with radioisotopes or dyes, and/or after linkage to drug or drug-loaded nanocarriers, LDLR-targeting peptides could be respectively used in molecular imaging for tumor diagnosis (e.g., MRI, PET) and surgical tumor resection (near-infrared fluorescence guided-surgery), and/or in therapeutics.

## Results

### LDLR is a promising candidate for human PDAC targeting

**Fig. 1:**
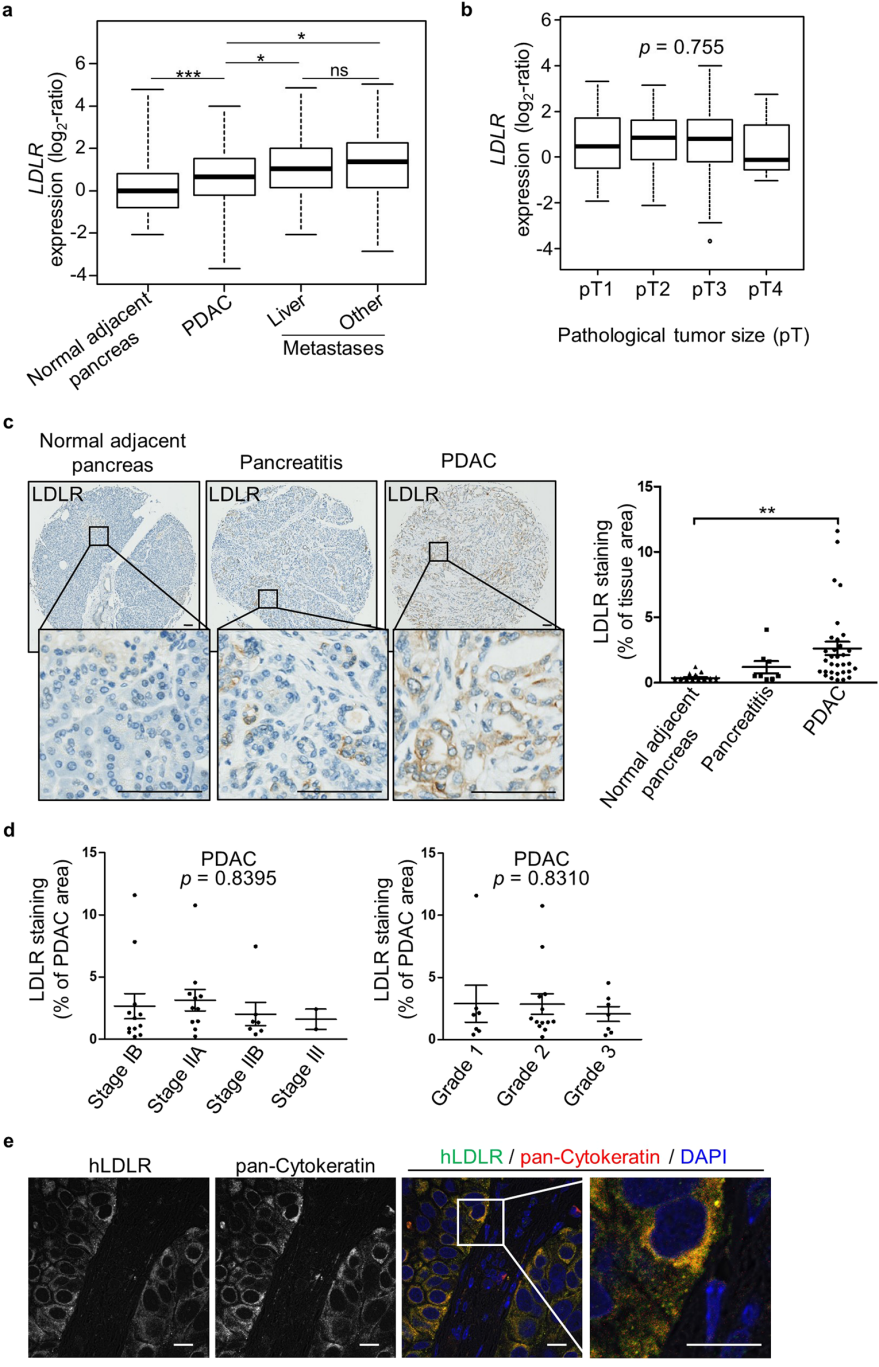
Human LDLR is a promising candidate for PDAC targeting. Box-and-whisker plots of human *LDLR* expression (**a**) in normal adjacent pancreas (*n* = 96 biologically independent samples), and in primary (*n* = 728 biologically independent samples) and metastatic PDAC (*n* = 76 biologically independent samples, including 35 liver metastases); **b** in primary PDAC according to pathological tumor size (pT) (pT1: *n* = 17, pT2: *n* = 61, pT3: *n* = 294 and pT4: *n* = 11 biologically independent samples). **a**, **b** Median values of *LDLR* expression in PDAC and metastasis samples were compared to that of normal adjacent pancreas samples and expressed as log_2_-ratio. **c** Representative immunohistochemistry (IHC) and automatic quantitation of LDLR in pancreatitis (acute: *n* = 1 and chronic: *n* = 6 biologically independent samples), PDAC (*n* = 32 biologically independent samples) and normal adjacent pancreas (*n* = 22 biologically independent samples) from patient pancreatic tissue microarray. Mean value ± s.e.m. of two independent tissue cores from each patient sample was illustrated. LDLR IHC images: ×6.8 and ×20 magnification, scale bar: 100 µm. **d** LDLR levels in patient PDAC according to tumor stage (left, *n* = 32 biologically independent samples) and grade (right, *n* = 27 biologically independent samples). **a**, **d** One-way ANOVA with post-hoc Tukey HSD test, ns: no significant difference, *p < 0.05, **p < 0.01, ***p < 0.001. **e** Representative confocal microscopy images of BxPC-3 orthotopic xenograft sections representing co-staining of human LDLR (in green color) and epithelial tumor cell marker (pan-Cytokeratin in red color). ×60 magnification, scale bar: 10 µm. An enlarged merge image of the indicated part is provided in the inset box. *n* = 3 mice/group.

We have previously shown that PDAC modifies its metabolic requirements, making this tumor highly reliant on LDL-derived cholesterol uptake through LDLR. To investigate whether LDLR is a suitable candidate for targeting PDAC in patients, we first performed an in silico analysis of the *LDLR* mRNA expression profile in primary and metastatic pancreatic tumor samples (*n* = 728 and *n* = 76, respectively) compared to normal adjacent pancreas (*n* = 96). The merged genomic dataset, established from fifteen published expression datasets of PDAC (Supplementary Table S1), revealed a significant increase in *LDLR* transcripts in primary PDAC (*p* = 7.42 × 10^−4^) and metastases regardless of their location (*p* = 1.40 × 10^−4^ (liver) and *p* = 1.02.10^−4^ (other)), as compared to normal adjacent pancreas (Fig. 1a). Interestingly, *LDLR* levels are higher in metastases than in primary PDAC, and showed no relation to either primary tumor size (*p* = 0.755, Fig. 1b) or American Joint Committee on Cancer stage defining anatomical extent of pancreatic cancer (*p* = 0.813, Supplementary Fig. 1a). We did however note that *LDLR* expression in primary tumors, whatever their stage, was most often higher compared to that in normal adjacent pancreas (Supplementary Fig. 1a).

Next, LDLR immunohistochemistry on pancreatic tissue microarray samples revealed a significantly higher level of positive LDLR staining in primary tumors, regardless of stage and degree of differentiation (i.e., grade), compared to normal adjacent pancreas (95% confidence interval: −3.875, −0.6477) (Fig. 1c, d). This high LDLR staining was also found in orthotopic PANC-1, MIA PaCa-2, and BxPC-3 xenografts (Fig. 1e and Supplementary Fig. 1b), and more specifically in tumoral epithelial cells, as validated by the co-localization of LDLR with pan-Cytokeratin epithelial marker (Fig. 1e).

Together, these results show that LDLR, highly represented in the epithelial compartment of patient’s PDAC irrespective of tumor size, stage and aggressiveness, constitutes a powerful candidate for tumor-specific targeting.

### Internalization and intracellular trafficking of LDLR–ligand complexes in murine pancreatic cancer cells

**Fig. 2:**
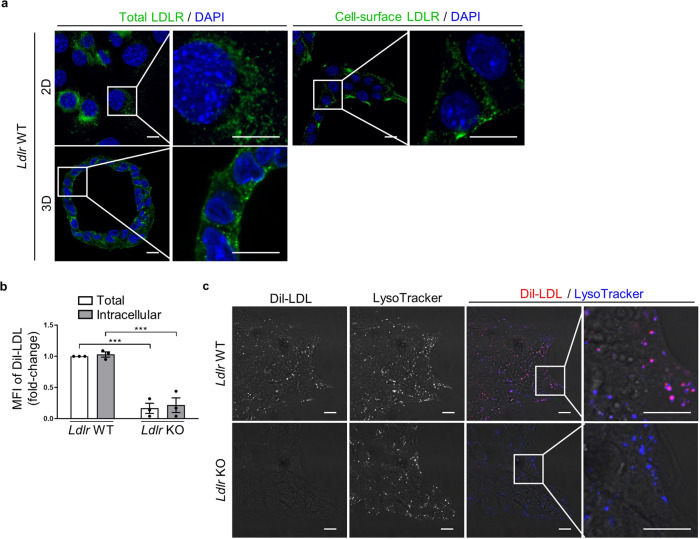
LDLR-dependent internalization and trafficking of LDL toward lysosomal compartment in pancreatic cancer cells. **a** Representative confocal microscopy images of total and/or cell surface LDLR (in green color) in 2D and 3D *Ldlr* WT cells. ×60 magnification, scale bar: 10 µm. An enlarged merge image of indicated part is provided in inset box. *n* = 2 independent experiments. **b** Flow cytometry assessment of total DiI-LDL (i.e., bound and internalized) in *Ldlr* WT and KO PK4A cells and of intracellular DiI-LDL in cells previously treated with an acid solution. Data are represented as fold-change ± s.e.m. of the mean of fluorescence intensity (MFI) of DiI-LDL and are expressed relative to value obtained in *Ldlr* WT cells and arbitrarily set to 1. One-way ANOVA with post-hoc Tukey HSD test, ***p < 0.001. *n* = 3 independent experiments. **c** Representative confocal microscopy images of DiI-LDL (in red color) and LysoTracker blue (i.e., late acidic endosome and lysosome marker) in live *Ldlr* WT and KO PK4A cells. x60 magnification, scale bar: 10 µm. An enlarged merge image of indicated part is provided in inset box. *n* = 3 independent experiments.

To assess whether LDLR has the key features required for optimal delivery of LDLR-targeting conjugate, we examined cell membrane internalization and intracellular trafficking of its natural ligand, LDL, in pancreatic cancer cells isolated from tumor of spontaneous KIC mouse model (i.e., PK4A cells^16^). In two-dimensional (2D) monolayer cells and three-dimensional (3D) organoid models, LDLR was located in the cytoplasm and at the cell surface of *Ldlr* wild-type (WT) PK4A cells (Fig. 2a), while no staining was observed in *Ldlr* knock-out (KO) PK4A cells generated by the CRISPR/Cas9 system (Supplementary Fig. 2a, b). Using flow cytometry, we showed that fluorescent LDL (DiI-LDL) was fully internalized by *Ldlr* WT PK4A cells, since no difference could be detected between total (i.e., bound and internalized DiI-LDL) and intracellular DiI-LDL levels (Fig. 2b and Supplementary Fig. 3a, b). As expected, LDL internalization was drastically reduced in *Ldlr* KO cells (Fig. 2b), attesting that LDLR is the main route of LDL internalization in PDAC cells. Furthermore, DiI-LDL was efficiently delivered to late endosome/lysosome, as showed by its co-localization with a specific marker of these endocytic vesicles (i.e., lysotracker) (Fig. 2c).

These findings attest that cell-surface LDLR is fully functional in KIC-derived PDAC cells with regards to the endocytosis of its natural ligand and its intracellular trafficking through the endosomal/lysosomal pathway.

### The LDLR-targeting conjugate is internalized by pancreatic cancer cells

**Fig. 3:**
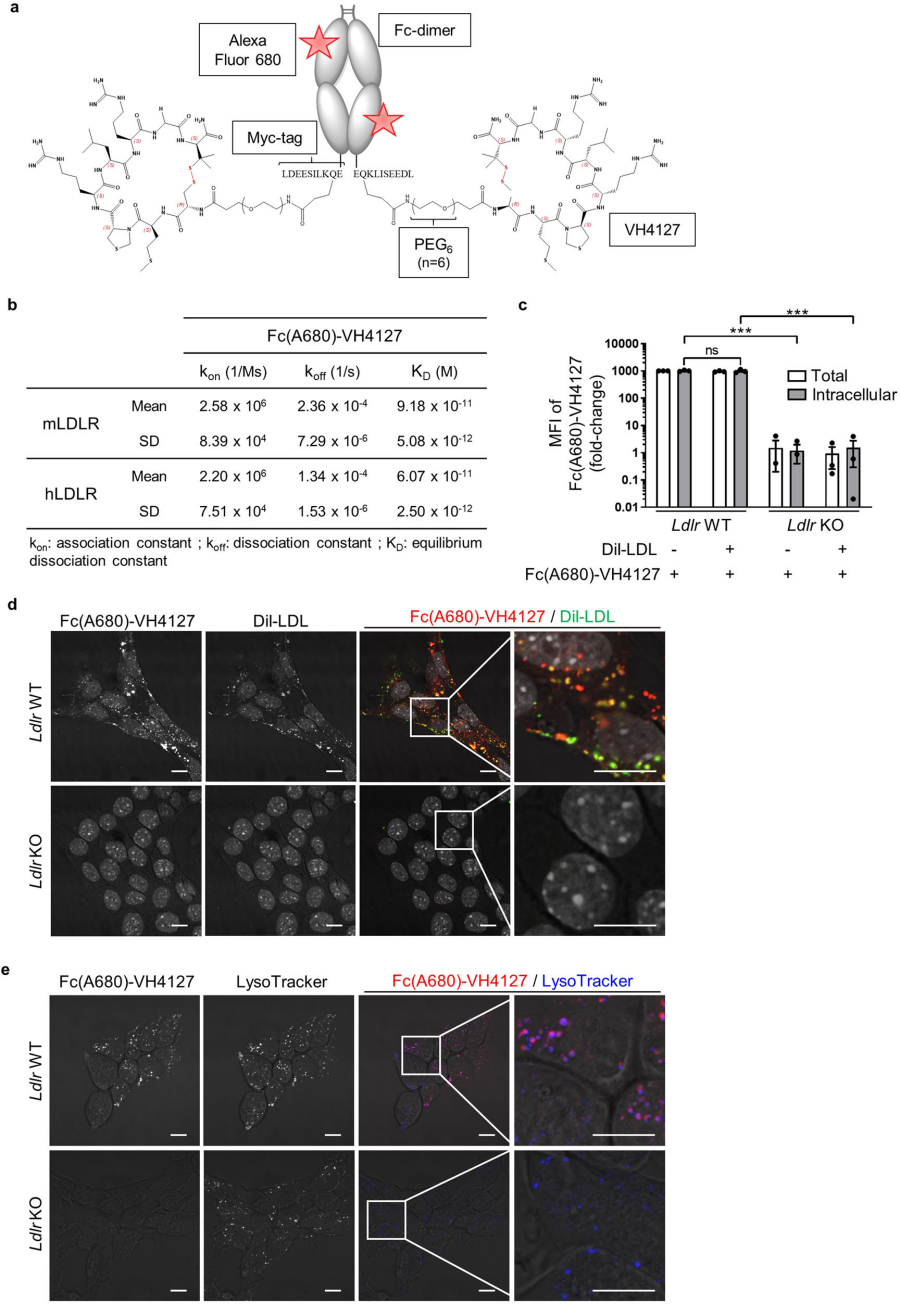
LDLR-dependent uptake of Fc(A680)-VH4127 conjugate by pancreatic cancer cells. **a** Schematic structure of the fluorescent Fc(A680)-VH4127 conjugate. **b** Kinetic parameters (*k*_on_ and *k*_off_) and equilibrium dissociation constant (K_D_) of Fc(A680)-VH4127 conjugate. Data are represented as mean ± standard deviation (SD). *n* = 3 independent experiments. **c** Flow cytometry analysis of total and internalized Fc(A680)-VH4127 in *Ldlr* WT and KO PK4A cells with or without DiI-LDL. Data are represented as fold-change ± s.e.m. of the mean of fluorescence intensity (MFI) of Fc(A680)-VH4127 expressed relative to value measured in *Ldlr* WT cells incubated without DiI-LDL and arbitrarily set to 1000. One-way ANOVA with post-hoc Tukey HSD test, ns: no significant difference, ***p < 0.001. *n* = 3 independent experiments. **d**, **e** Representative confocal microscopy images of Fc(A680)-VH4127 (pseudo-colored red) and DiI-LDL (pseudo-colored green) (**d**) or LysoTracker blue (**e**) in live *Ldlr* WT and KO PK4A cells. ×60 magnification, scale bar: 10 µm. An enlarged merge image of indicated part is provided in inset box. *n* = 3 independent experiments.

To enable the exploitation of cell surface LDLR for molecule delivery into pancreatic cancer cells, we used the cyclic VH4127 peptide, bearing non-natural amino acid residues, and which specifically binds to rodent and human epidermal growth factor (EGF) homology domain of LDLR (Supplementary Table S2). To prolong its exposure time to its target, the VH4127 peptide was conjugated via a polyethylene glycol spacer, containing 6 ethylene glycol units, to the C-terminus of dimeric Myc-tagged Fc fusion protein (Fig. 3a)^17^. The resulting bivalent Fc-VH4127 was finally covalently labeled with the Alexa Fluor 680 dye (A680) (i.e., Fc(A680)-VH4127), whose reduced autofluorescence is highly suitable for in vivo optical imaging purposes^18^. The surface plasmon resonance analysis revealed that the conjugation of VH4127 to Fc-based compound highly increases the affinity of the Fc(A680)-VH4127 conjugate for mouse and human LDLR compared to that of the VH4127 peptide alone (91.8 pM vs 51 nM and 60.7 pM vs 21.6 nM, respectively) (Fig. 3b vs Supplementary Table S2). The scramble Fc(A680)-VH4Sc conjugate^15^ was used as control conjugate.

We next evaluated whether the Fc(A680)-VH4127 conjugate enters into PDAC cells via LDLR-mediated endocytosis and is transported through the endosomal-lysosomal route. We showed that cell-surface Fc(A680)-VH4127 was completely internalized by *Ldlr* WT PK4A cells and interestingly, non-competitively with LDL (Fig. 3c). Consequently, the entry of DiI-LDL through LDLR was not hampered by the Fc(A680)-VH4127 (Supplementary Fig. 3b). The loss of LDLR led to a 1000-fold reduction in Fc(A680)-VH4127 internalization by PK4A cells (Fig. 3c). Interestingly, the residual quantity of Fc(A680)-VH4127 was similar to the amount of control conjugate (Fc(A680)-VH4Sc) internalized through a LDLR-independent route (Fig. 3c vs Supplementary Fig. 3c). Therefore, the Fc(A680)-VH4127 uptake is mainly governed by the LDLR-targeting peptide. We confirmed by confocal analysis that unlike the control conjugate, the Fc(A680)-VH4127 was taken up by cells just as the natural ligand, in a LDLR-dependent manner, and was efficiently delivered to the late endosome/lysosome compartment, as shown by its co-localization with lysotracker (Fig. 3d, e vs Supplementary Fig. 3d, e).

Overall, these data highlight that Fc(A680)-VH4127 conjugate specifically targets LDLR in pancreatic cancer cells and, as the natural ligand of LDLR, is properly internalized by endocytosis and delivered to lysosomes.

### The LDLR-targeting conjugate is selectively addressed and up-taken by PDAC in vivo

**Fig. 4:**
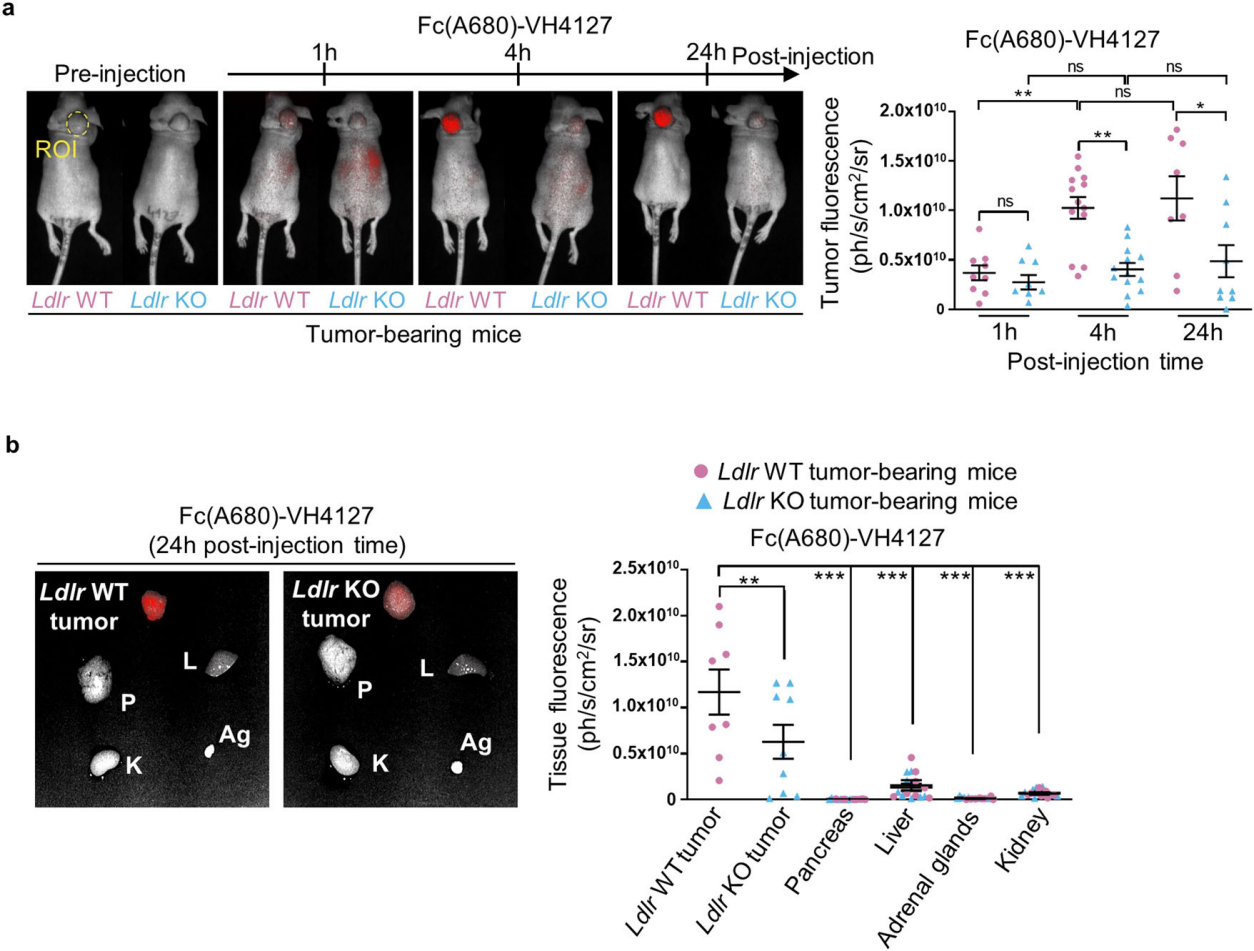
The Fc(A680)-VH4127 specifically targets subcutaneous pancreatic tumors. **a** Representative merged fluorescence and visible light images of *Ldlr* WT and *Ldlr* KO pancreatic tumors grown subcutaneously in immunodeficient mice. Fluorescence acquisitions were performed before intravenous injection (IV) of Fc(A680)-VH4127 conjugate (1 nmole/mouse) and 1, 4, and 24 h after. The kinetic of tumor-specific fluorescence of Fc(A680)-VH4127 (i.e., region of interest (ROI)) is illustrated and expressed as mean (ph/s/cm^2^/sr) ± s.e.m. **b** Representative merged fluorescence and visible light images of the tumors (*Ldlr* WT and *Ldlr* KO), pancreas (P), liver (L), adrenal glands (Ag) and kidney (K) acquired 24 h after administration of Fc(A680)-VH4127 conjugate. The tissue-specific fluorescence of Fc(A680)-VH4127 was illustrated and expressed as mean (ph/s/cm^2^/sr) ± s.e.m. **a**, **b** One-way ANOVA with post-hoc Tukey HSD test, ns: no significant difference, *p < 0.05, **p < 0.01, ***p < 0.001. *n* = 8–13 mice at each time-point.

PDAC targeting by Fc(A680)-VH4127 was first explored in athymic mice subcutaneously transplanted with *Ldlr* WT or *Ldlr* KO PK4A cells using real-time NIRF imaging. Fluorescence acquisitions of living tumor-bearing mice were performed following intravenous injection of either Fc(A680)-VH4127 or control conjugate. The quantitative analysis of fluorescence restricted to tumor area showed that Fc(A680)-VH4127 accumulation into *Ldlr* WT tumors reached a peak from 4 h and up to 24 h post-injection (Fig. 4a, left), that was twice that observed at 1 h post-injection. As expected, the *Ldlr* KO tumors, characterized as *Ldlr* WT tumors by prominent well-differentiated and dedifferentiated tumor cells areas (Supplementary Fig. 4a, b), showed no increase in Fc(A680)-VH4127 fluorescence (Fig. 4a), thus validating the LDLR-dependent accumulation of the conjugate in PDAC in vivo. The fluorescence values of the control conjugate (Fc(A680)-VH4Sc) on the other hand remained stable and substantially lower than those of LDLR-targeted conjugate (Supplementary Fig. 4c vs Fig. 4a) as it does not target *Ldlr* WT tumors. Hence, the observed accumulation of fluorescence in PDAC was specifically due to the VH4127 peptide.

We next characterized the tissue biodistribution profile of Fc(A680)-VH4127 and its control counterpart by ex-vivo fluorescence acquisitions at 4 h and 24 h post-injection. Despite tissue heterogeneity of LDLR levels in this tumor-bearing mouse model (Supplementary Fig. 4d), fluorescence quantitation revealed a selective accumulation of Fc(A680)-VH4127 in *Ldlr* WT tumors when compared to Fc(A680)-VH4127 values in all other healthy organs, including pancreas (Fig. 4b). Interestingly, at 24 h post-injection, this highest accumulation in *Ldlr* WT tumors was associated with the lowest levels in excretory organs (Fig. 4b vs Supplementary Fig. 4e), indicating highest tumor-selective targeting potential at this time-point. A slight accumulation of Fc(A680)-VH4127 was however detected in *Ldlr* KO tumors as compared to other healthy tissues, demonstrating that non-selective tumor-uptake also exists. Importantly, this residual signal is close to control conjugate signals measured in both tumors and in kidney independently of the LDLR status (Supplementary Fig. 4f).

These findings illustrate the high potential of the LDLR-targeting conjugate in PDAC targeting in vivo, an essential property for its consideration as a useful tool in imaging and therapeutic applications.

### The LDLR-targeting conjugate differentiates advanced PDAC from chronic pancreatitis

Next, we further investigated the potential of the LDLR-targeting conjugate for use as a clinical tool aiming to specifically target stroma-rich PDAC rather than pancreatic diseases, such as mass-forming chronic pancreatitis (CP). To this end, we used spontaneous KIC mice that faithfully recapitulate the molecular and histopathological features of human disease^11^, and their WT littermate counterparts (KI mice: LSL-Kras^G12D^; Ink4a/Arf^fl/fl^) that we submitted or not to CP induction (KI + CP or KI mice, respectively) (Supplementary Fig. 5a). In inflammatory pancreas from KI + CP mice, the LDLR staining was restricted to few altered acinar structures, while no staining was observed in healthy pancreas (Supplementary Fig. 5b). In contrast, as previously reported^10^, all tumoral glands in advanced PDAC were LDLR positive (Supplementary Fig. 5b). These differences in LDLR levels were confirmed by immunoblot analysis (Supplementary Fig. 5c). Hence, we considered that KIC and KI + CP mice are suitable preclinical models to evaluate LDLR-targeting conjugate ability to discriminate PDAC from CP, which still remains a diagnostic challenge in imaging.

**Fig. 5:**
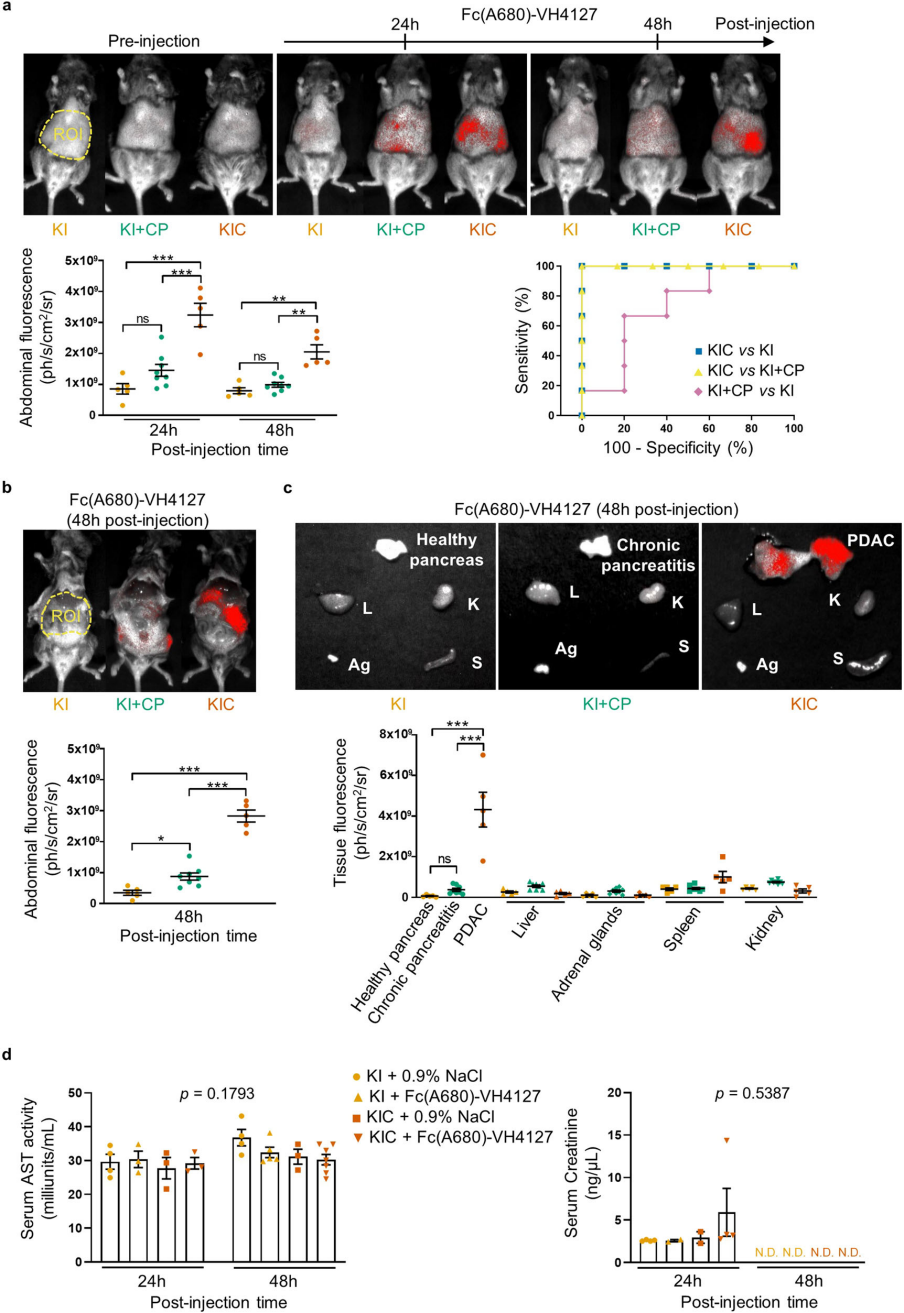
The Fc(A680)-VH4127 conjugate discriminates advanced PDAC from inflammatory and healthy pancreas without causing hepatic and renal damages. **a** Representative merged fluorescence and visible light images of control (KI), induced-chronic pancreatitis (KI + CP), and spontaneous PDAC-bearing (KIC) mice before, 24 and 48 h after retro-orbital IV injection of the LDLR-targeting conjugate (1 nmole/mouse). Quantification of Fc(A680)-VH4127 fluorescence in abdominal region (bladder excluded) of each experimental group is illustrated (left graph). Receiver operating characteristic (ROC) curve established from Fc(A680)-VH4127 fluorescence values obtained at 48 h post-injection in each experimental group is shown in right graph. **b**, **c** Representative merged fluorescence and visible light images of KI, KI + CP, and KIC mice after euthanasia and laparotomy (**b**) and of excised pancreas (healthy, inflammatory or tumoral), liver (L), kidney (K), adrenal glands (Ag) and spleen (S) (**c**), performed 48 h after Fc(A680)-VH4127 injection. Quantification of Fc(A680)-VH4127 fluorescence in abdominal region (bladder excluded) (**b**) or organ area (**c**) is illustrated. **a**, **c** Data are expressed as mean (ph/s/cm^2^/sr) ± s.e.m. One-way ANOVA with post-hoc Tukey HSD test, ns: no significant difference, *p < 0.05, **p < 0.01, ***p < 0.001. *n* = 5 KI and KIC mice and *n* = 8 KI + CP mice. **d** Aspartate aminotransferase (AST) activity and creatinine levels in serum of KI and KIC mice measured 24 and 48 h after injection of Fc(A680)-VH4127 (1 nmole/mouse) or vehicle (0.9% NaCl, equivalent volume). One-way ANOVA with post-hoc Tukey HSD test, N.D.: not detectable. *n* = 3–7 mice/group.

Twenty-four and 48 h after intravenous injection of Fc(A680)-VH4127 to advanced PDAC-bearing KIC mice and age-matched KI + CP and KI mice, we observed a higher fluorescence signal in the abdominal part of KIC mice than in that of KI and KI + CP mice at any time post-injection (Fig. 5a, top and bottom left panels). The corresponding receiver operating characteristic curve illustrates the diagnostic ability of Fc(A680)-VH4127 to specifically and accurately discriminate PDAC from CP (KIC vs KI + CP, threshold: 1.33 × 10^9^ ph/s/cm^2^/sr, area under the curve (AUC) = 1 and *p* = 0.003965), and from healthy pancreas (KIC vs KI, threshold: 1.27 × 10^9^ ph/s/cm^2^/sr, AUC = 1 and *p* = 0.006192) with a sensitivity and a specificity of 100% (Fig. 5a, bottom right panel). After abdominal laparotomy, we validated that the Fc(A680)-VH4127 signal perfectly matches with tumor location (Fig. 5b).

Next, we investigated the biodistribution of Fc(A680)-VH4127 in malignant and healthy tissues, showing distinct LDLR levels (Supplementary Fig. 6a, b), from the three experimental groups. Like tumors, liver, and adrenal glands show the highest LDLR rates with a LDLR localization restricted to the cell surface of hepatocytes and adrenal gland cells, as revealed by immunohistochemistry analysis (Supplementary Fig. 6c). Nevertheless, Fc(A680)-VH4127 was shown to only accumulate in PDAC at 48 h post-injection and not in liver and adrenal glands, nor in other tissues (Fig. 5c). The Fc(A680)-VH4127 fluorescence was respectively 12- and 24-fold higher in PDAC than in inflammatory or healthy pancreas. Using immunofluorescence staining, we also provided further insight into the spatial location of Fc/LDLR double positive cells within PDAC sections of KIC mice injected with Fc(A680)-VH4127 (Supplementary Fig. 6d).

In regards with healthy tissues, lack of significant accumulation of the conjugate was also noted at an earlier post-injection time (24 h vs 48 h) (liver: *p* = 0.0739, kidney: *p* = 0.1959, adrenal glands: *p* = 0.2124, and spleen: *p* = 0.0207) (Supplementary Fig. 6e). These results were consistent with the preclinical toxicology study showing no adverse effects of the conjugate on hepatic and renal functions of control and tumor-bearing mice (Fig. 5d). Indeed, no significant increase in serum aspartate aminotransferase activity and creatinine levels was detected 24 h and 48 h post-injection of the conjugate as compared to vehicle injection. However, we noted the presence of creatinine in serum collected at 24 h, while it became undetectable at 48 h independently of the compound injected (conjugate or vehicle) and whatever the mice genotype (KI and KIC). Similarly, no sign of liver and kidney damages was revealed by histological examination (Supplementary Fig. 7).

Together these preclinical data demonstrate that the Fc(A680)-VH4127 conjugate detects PDAC with high levels of desmoplasia and reliably discriminates chronic pancreatitis from pancreatic tumor, without causing any hepato- and nephrotoxicity.

### Targeting pancreatic tumor cells disseminated in liver with Fc(A680)-VH4127

**Fig. 6:**
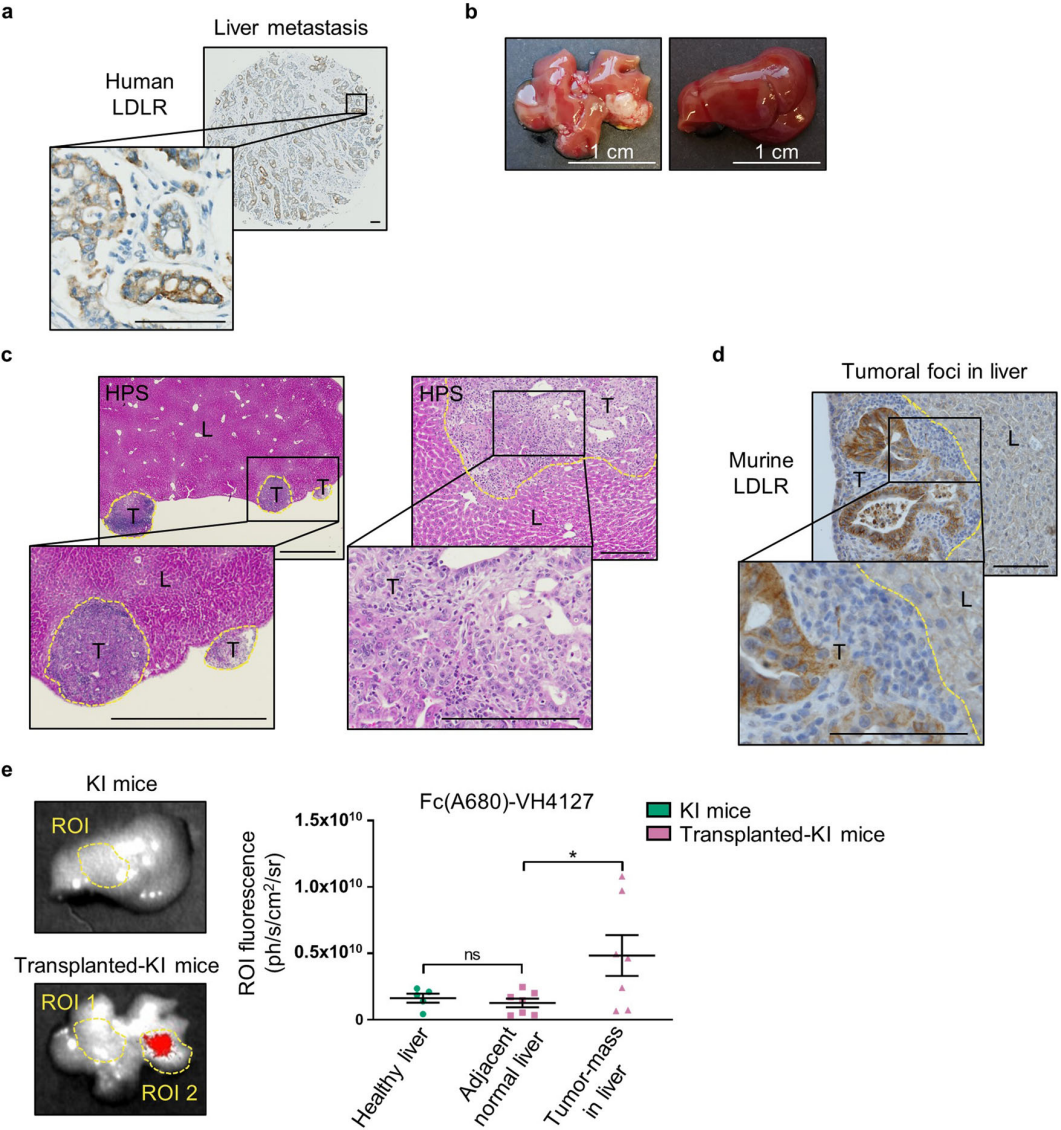
The Fc(A680)-VH4127 conjugate detects pancreatic tumor mass in liver. **a** Representative IHC of LDLR in liver metastasis from PDAC resected patient. ×6.8 and ×20 magnification, scale bar: 100 µm. *n* = 2 independent samples. **b** Representative image of PDAC foci in liver from KI mice transplanted with PK4A cells (left) and of healthy liver from KI mice (right). **c**, **d** Representative Haematoxylin Phloxine Saffron (HPS) (**c**) and LDLR (**d**) staining of PDAC foci (T) in liver (L) of transplanted-KI mice, delimited by yellow dots. An enlarged image of indicated part is provided in inset box. *n* = 5 mice. **c** Left panel: ×2 magnification, scale bar: 1000 µm; right panel: ×10 magnification, scale bar: 500 µm. **d** ×20 magnification, scale bar: 100 µm. **e** Representative merged fluorescence and visible light images of excised healthy- and tumoral liver from KI and transplanted-KI mice, respectively, obtained 48 h after retro-orbital IV injection of Fc(A680)-VH4127 (1 nmole/mouse). Quantification of ROI-specific Fc(A680)-VH4127 signal relative to healthy pancreas from KI mice and to tumoral foci and adjacent normal liver from transplanted-KI mice. Data are expressed as mean (ph/s/cm^2^/sr) ± s.e.m. One-way ANOVA with post-hoc Tukey HSD test, ns: no significant difference, *p < 0.05. *n* = 5 and *n* = 7 KI and transplanted-KI mice, respectively.

We showed that LDLR-targeting conjugate specifically targets primary tumors in induced and spontaneous PDAC mouse models. The next step was to evaluate the ability of Fc(A680)-VH4127 to target secondary tumors localized in liver, which is the most frequent metastatic site in PDAC patients^19^. This assumption is supported by IHC analysis showing high LDLR staining in liver metastatic cancer cells from patient PDAC (Fig. 6a), confirming the gene expression data (Fig. 1a).

Intra-hepatic implantation of PK4A cells in KI mice led to the development of multiple tumoral foci of various size in the liver (ranging from 2 to 15 mm) (Fig. 6b, c), in which LDLR is localized both in tumoral foci and in the surrounding hepatocytes (Fig. 6d). Despite this dual LDLR location, in ex-vivo liver we observed that the Fc(A680)-VH4127 was able to discern the hepatic tumor cell niches from surrounding hepatocytes. Indeed, a 3.8- and 3-fold increase in conjugate fluorescence was observed in hepatic tumor mass as compared to the fluorescence measured in adjacent liver of PK4A transplanted-KI mice or in healthy liver from KI mice, respectively (Fig. 6e).

These data demonstrate the higher avidity of LDLR-targeting conjugate for pancreatic tumor cells disseminated within the liver than for the surrounding hepatocytes, and offer a new clinical perspective for the use of the LDLR-targeting peptide combined to radioisotope for detecting and/or treating PDAC metastases using PET imaging.

## Discussion

Development of targeted delivery systems for the PDAC in order to improve both diagnostic imaging modalities with imaging probes, as well as efficiency of anti-cancer drugs, is expected to improve patient outcome. One main challenge has been identifying a tumor cell-surface target that would allow specific docking for tumor imaging or specific-uptake and thereby intracellular delivery of therapeutic drugs. Here our data, demonstrating the efficient and specific binding and internalization of Fc(A680)-VH4127 conjugate by PDAC cells, highlight the LDLR as a reliable candidate through which pancreatic tumors at both primary and metastatic sites may be targeted.

In our study, gene expression meta-analysis of human PDAC samples showed increasing levels of *LDLR* expression with tumor progression. Interestingly, LDLR protein levels did not change with anatomical extent or aggressiveness of PDAC from patients, suggesting that LDLR-targeting peptides may be useful to diagnose and treat all PDAC. Furthermore, their use could be extended to other types of cholesterol-addictive tumors, such as glioblastoma and breast tumors, which also rely on LDL uptake to satisfy their needs in cholesterol and enable their proliferation^20–22^.

The Fc(A680)-VH4127 conjugate used in this work shows remarkable properties such as bivalent binding to mouse and human LDLR with excellent affinity (91.8 pM and 60.7 pM, respectively), high proteolytic stability of the VH4127 peptide ligand, and extended serum half-life due to the Fc backbone^15,23^. In vitro, this conjugate was mostly taken up by PDAC cells through the LDLR abundantly present at the cell surface and fully functional. When administered to tumor-bearing mice, the conjugate also weakly accumulated in *Ldlr* KO tumors, at much lower levels compared to in *Ldlr* WT tumors, yet considerable as compared to healthy organs. Several hypotheses can account for this moderate LDLR-independent uptake of Fc(A680)-VH4127 conjugate in *Ldlr* KO tumors. In tumors harbouring a *KRAS* mutation, macropinocytosis is a non-specific macro-molecule uptake mechanism, essential to supply amino acids to nutrient deprived-PDAC cells^24–27^. Hence, Fc(A680)-VH4127 penetration at 24 h post-injection might result from an activation of macropinocytosis in *Ldlr*-depleted PDAC, a process that serves also as entry route for nanomedicines, including protein-drug conjugates and drug-loaded nanoparticles^28^. Tumor-accumulation of Fc(A680)-VH4127 in *Ldlr* KO PDAC could also involve the passive enhanced permeability and retention (EPR) effect, which results from tumor vasculature leakiness favouring entry of macromolecules^29^. However, the efficiency of EPR-based tumor accumulation tends to be very limited in stroma-rich tumors like PDAC^30^. Indeed, the prominent stroma impairs perfusion of tumor compartments by compressing the tumor microvessels. Finally, we cannot rule out that in the absence of LDLR the Fc(A680)-VH4127 conjugate could bind to EGF precursor homology domain of other proteins, including other members of the LDLR family^31^ that might be present in PDAC.

Interestingly, using the spontaneous PDAC mouse model, we demonstrated that the Fc(A680)-VH4127 conjugate has the ability to bypass the stromal barrier, which to date has made the PDAC extremely hard-to-reach with imaging tracers and/or conventional chemotherapies^32,33^. Indeed, despite PDAC from KIC mice exhibit tumor microenvironment constraints resembling those observed in humans^11^, such as desmoplasia composed of non-cellular components (i.e., extracellular matrix) and stromal cells^34^, these latter do not seem to impede the uptake of the LDLR-targeting conjugate by the tumor cells. Other important findings are the higher accumulation of Fc(A680)-VH4127 in PDAC at 24 and/or 48 h post-injection compared to healthy tissue (i.e., liver and adrenal glands) expressing similar LDLR levels to those in tumors, as well as the lack of hepato- and nephrotoxicity of the conjugate, as demonstrated by the toxicity study. Interestingly, the LDLR-targeting conjugate has the ability to discern liver disseminated PDAC cells from surrounding hepatocytes at 48 h post-injection. At these time-points, accumulation of the LDLR-targeting conjugate within healthy organs appears insignificant, while it remains substantial within the tumor. This could be explained by the high metabolic demand for LDL-cholesterol that pancreatic cancer cells might satisfy by developing adaptive mechanisms, such as increased LDLR-mediated endocytosis and/or LDLR-recycling^35^. An in-depth analysis of the dynamics of these processes in PDAC cells will help address this question.

Our findings have clearly demonstrated the good diagnostic performance of the fluorescent LDLR-targeting peptide in detecting tumor mass at metastatic sites, especially in excised liver. Further experiments on alive animals are necessary to investigate the feasibility of detecting liver metastasis by near-infrared imaging. Thus, our study uncovers the tissue penetration depth limit of near-infrared imaging, which can be exclusively used as real-time navigation modality during laparoscopic surgery to localize primary and secondary tumors and achieve clear resection margins. Moreover, to model the hepatic dissemination of pancreatic cancer, we deliberately performed intra-hepatic injection of murine pancreatic cancer cells so as not to occult metastases signal from that of primary tumor. However, these limitations are waived when using radioisotopes conjugated to LDLR-targeting peptide in PET imaging modalities, endowed with unlimited depth penetration and its high sensitivity. We also highlight the ability of the Fc(A680)-VH4127 to distinguish PDAC-bearing mice from mice suffering from chronic pancreatitis, a well-known challenge. Indeed, effective imaging modalities able to discriminate CP and PDAC which share similar symptoms at diagnosis^36^, as well as to depict small liver and peritoneal metastases^37^ which account for most PDAC-associated deaths, are currently lacking. The use of LDLR-targeting peptides conjugated to radiotracers may prove a useful PET/CT or MRI imaging tools allowing clinicians to make the accurate pre-operative diagnosis and thereby selecting the most appropriate treatment for each patient, as well as the non-invasively tracking of metastases evolution thus avoiding repeated biopsies. Indeed, up to 40% of patients assessed as resectable following preoperative diagnosis are then judged unresectable upon laparoscopic exploration, due to an insufficient diagnostic performance of current imaging methods. By its ability to selectively accumulate in tumor and thereby provide substantial contrast against healthy pancreas, other applications can be assigned to LDLR-targeting peptides, such as intraoperative near-infrared fluorescence imaging to assist PDAC surgical treatment, as already demonstrated in PDAC xenograft mouse model^14^, and tumor-specific drug delivery. LDLR-targeting peptides, known for their ability to cross the blood-brain barrier^38^, have been used to functionalize paclitaxel-loaded nanoparticles^39^ to target glioma. This targeted-delivery of nanoparticules thus enhances the anti-tumoral effect of paclitaxel and survival of glioma-bearing mice. Our in vitro results show that LDLR-targeting conjugate enters into PDAC cells through LDLR mediated-endocytosis before trafficking to the late endosome/lysosome pathway, emulating the natural ligand LDL. These properties make LDLR-targeting peptides promising candidates for conveying anti-cancer drugs to the lysosome for subsequent acidic pH-mediated release^40^.

Altogether, this proof-of-concept study, highlighting the powerful potential of LDLR-targeting peptides as vehicles for nuclear imaging probes and/or drugs, offers hopeful perspectives both in medical imaging for pre-operative diagnosis and in cancer treatments through fluorescence guided-surgery and targeted-drug delivery.

## Methods

### *LDLR*-based genomic meta-analysis in human pancreatic samples

The merged genomic dataset, established from 15 publicly available mRNA expression datasets (Supplementary Table S1), is composed of 728 PDAC, 76 metastases (including 36 liver metastases), and 96 normal adjacent pancreatic samples. Other metastatic samples come from the peritoneum (29%), the lymph nodes (22%) and the lung (20%). This in silico meta-analysis was thus based upon data from published studies for which the informed patients’ consent to participate and the ethics approval from the institutional review board were already obtained by the authors. The data analysis required pre-analytic processing. First, we separately normalized each DNA microarray-based dataset: individual Affymetrix dataset was normalized using robust multichip average with the non-parametric quantile algorithm for the raw Affymetrix data (Bioconductor and associated-packages, R software), while non-Affymetrix datasets were normalized with quantile normalisation procedure. Then, Agilent or Affymetrix hybridization probes were mapped across different technological platforms (Stanford Online Universal Resource for Clones and ESTs (SOURCE, https://source-search.princeton.edu/), NCBI EntrezGene (ftp://ftp.ncbi.nlm.nih.gov/gene/, Homo sapiens gene information db, release from 04/27/2017) and Affymetrix NetAffx Annotation files (www.affymetrix.com; release from 01/12/2008). When multiple probes mapped to the same gene, we retained the most variant probe in a particular dataset. For the RNA-seq data, we used the available normalized data. We then log_2_-transformed the normalized genomics data. Subsequently, we corrected the fifteen studies for batch effects using z-score normalization. Briefly, for each separate *LDLR* expression value in each study, we subtracted the mean value of the gene in that dataset divided by its standard deviation, mean and standard deviation only being measured on primary tumor samples. Next, we represented the median value of *LDLR* expression in PDAC or metastases samples as a function of the median value of *LDLR* expression in normal adjacent pancreas samples.

### Quantitative assessment of LDLR in pancreatic tissue microarray (TMA)

LDLR IHC staining of formalin-fixed and paraffin-embedded (FFPE) section of pancreatic TMA (PA2081c, US Biomax, USA) was fully automated process (Discovery XT Automated slide staining system, Ventana Medical System, USA). Briefly, after the deparaffinization and the antigen retrieval steps (Citrate-based buffer, pH 6.0), TMA section was incubated with blocking solution and then with goat anti-human LDLR antibody (1/150, AF2148, R&D Systems) for 1 h at 37 °C. OmniMap anti-goat HRP (760-4647, Ventana Medical System, USA) was applied on tissue section and then 3,3-diaminobenzidine (DAB) in presence of H_2_O_2_ to reveal LDLR staining. Finally, slide was counterstained with hematoxylin (CS70030, Dako), post-counterstained with bluing reagent before to be dehydrated, cleared and mounted with coverslip. LDLR quantification was performed with Calopix software (Tribvn Healthcare, France). Briefly, TMA cores were individualized and identified on scanned slide before to be segmented according to luminance, hue and mathematical morphological criteria. Then, an automated quantification of the LDLR stained surface was realized using the morphometry algorithm.

### Synthesis and labeling of the bivalent Fc(A680)-VH4127 and Fc(A680)-VH4Sc conjugates

The VH4Sc^15^ and VH4127 peptides (Supplementary Table S2), were synthesized using an automated micro-wave assisted Fmoc/tBu solid phase peptide synthesis strategy. A rink amide AM resin and hexafluorophosphate azabenzotriazole tetramethyl uronium (HATU) were selected as solid support and coupling reagent, respectively. A polyethylene glycol spacer composed of 6 ethylene glycol units (PEG_6_) and containing an Fmoc-protected amine (Fmoc-N-amido-PEG_6_-acid) was manually conjugated to the N-terminus of each synthetized peptide using COMU as coupling reagent. Then, the Fmoc protection of each peptide was removed to get a free N-terminal amine. Cleavage of the peptides from their solid support was performed by treatment for 2 h with a scavenger cocktail composed of TFA/DTT/H2O/TIS (94/2/2/2). The crude linear peptides obtained, H-PEG_6_-VH4127-NH_2_ and H-PEG_6_-VH4Sc-NH_2_, were precipitated with cold ethyl ether and freeze-dried without further purification. Cyclization of the peptides was directly performed by formation of a disulfide bridge between the two cysteins or analog thereof in positions 1 and 8 of the peptides using potassium ferricyanide (K3Fe(CN)_6_) as a mild oxidizing agent in the pH 8–9 range. After purification by preparative reversed-phase HPLC (RP-HPLC) using a C18 column, the fractions corresponding to the expected product with a purity assessed by analytical RP-HPLC above 95% were pooled and freeze-dried to get write powders. The purity and the identity of each synthetized peptide were respectively determined by analytical RP-HPLC and ESI mass spectrometry. Then, the cyclic H-PEG_6_-VH4127-NH_2_ and H-PEG_6_-VH4Sc-NH_2_ precursors were enzymatically conjugated to Fc-Myc fusion protein by the microbial transglutaminase (Zedira, Darmstadt, Germany). The Fc-Myc fusion protein, expressing the Myc-tag (EQKLISEEDL) at the C-terminus, was produced in Expi293 cells transiently transfected with the pINFUSE-IgG1-Fc_2_ plasmid vector (InvivoGen, San Diego, USA). Fc-based compounds were purified with Protein A GraviTrap gravity-flow columns (GE Healthcare) according to the manufacturer’s instructions. After conjugation and purification, the Fc-VH4127 and Fc-VH4Sc were concentrated and buffer exchanged with PBS (1×, pH 7.4) using Amicon Ultra centrifugal filter units (MWCO 30 kDa, Merck, Darmstadt, Germany). The amount of purified Fc-VH4127 or Fc-VH4Sc conjugates was assessed by measuring its absorbance at 280 nm, while the Peptide/Antibody Ratio (PAR), which corresponds to the number of conjugated-peptide H-PEG_6_-VH4127-NH_2_ or H-PEG_6_-VH4Sc-NH_2_ per Fc fragment molecule, was determined by MALDI-TOF mass spectrometry. This ratio was calculated by dividing the mass increment, calculated from MALDI-TOF mass spectra collected before and after conjugation of the H-PEG_6_-VH4127-NH_2_ or H-PEG_6_-VH4Sc-NH_2_ peptide, by their respective molecular weights. Experimental PAR was estimated to 2.0 for Fc-VH4127 and to 1.6 for Fc-VH4Sc conjugates. Finally, both conjugates were labeled with Alexa Fluor 680 (A680) SAIVI antibody labelling kit (Life Technologies) according to the manufacturer’s instructions. The protein concentration and the degree of A680 labelling (DOL) of the final Fc(A680)-VH4127 and Fc(A680)-VH4Sc conjugates were determined by measuring the absorbance at 280 nm and 680 nm, respectively, using a Nanodrop 1000 spectrophotometer (Thermo Fisher Scientific). Experimental A680 DOL was estimated to 1.7 for the Fc(A680)-VH4127 and to 1.6 for Fc(A680)-VH4Sc conjugate.

### Surface plasmon resonance (SPR)

After immobilization of His-tagged recombinant mouse and human LDLR (SinoBiological (Beijing, China) on a NiHC1000m sensor chip (Xantec, Dusseldorf, Germany), VH4127 (10–160 nM) and Fc(A680)-VH4127 (1–16 nM) were injected over flowcell by using the single-cycle kinetic method. Double-subtracted sensorgrams were fitted globally with the 1 :1 Langmuir model using Biacore T200 Evaluation version 3.2 software.

### PDAC cell models

Stable *Ldlr* KO cells were generated from PDAC cells derived from spontaneous tumors from KIC mice (*LSL-KRas*^*G12D*^*; Ink4a/Arf*^*fl/fl*^*; Pdx1-Cre*)^11^, and previously named PK4A cells^16^. *Ldlr* CRISPR/Cas9 KO and *Ldlr* Homology-Directed Repair (HDR) plasmids (sc-421408 and sc-421408-HDR, Santa Cruz) were co-transfected in PK4A cells using Lipofectamine 2000 (Invitrogen). Positive *Ldlr* KO PK4A cells expressing red fluorescent protein reporter were isolated by fluorescence-activated cell-sorting (FACS) and then cultured in 1 µg/mL of puromycine (Gibco). *Ldlr* WT PK4A cells were transfected with the control CRISPR/Cas9 plasmid containing a non-targeting sgRNA (sc-418922, Santa Cruz). Cells were cultivated in 2D in high glucose DMEM with GlutaMax (Gibco) supplemented with 10% fetal bovine serum (FBS) (BioSera), and in 3D with matrigel in a specific medium^41^. PANC-1 (CRL-1469), MIA PaCa-2 (CRL-1420), and BxPC-3 (CRL-1687) cells were purchased from American Tissue Cell Culture (ATCC, USA). PANC-1 and MIA PaCa-2 cells were cultivated in high glucose DMEM with GlutaMax (Gibco) supplemented with 10% FBS (BioSera), while BxPC-3 cells were cultivated in advanced RPMI medium 1640 with GlutaMax (Gibco) supplemented with 10% FBS. All cell lines, tested negative for mycoplasma contamination (Mycoalert Plus mycoplasma detection kit, Lonza, LT07-318) and authenticated using short tandem repeat analysis, as described in Capes-Davis *et al*.^42^, were cultivated at 37 °C in a humidified atmosphere (21% O_2_ and 5% CO_2_).

### Uptake and intracellular trafficking of fluorescent LDL and conjugates

10,000 *Ldlr* WT or KO PK4A cells were grown on glass coverslips for 48 h in DMEM with GlutaMax-10% FBS. Then, cells were incubated with DiI-LDL (20 µg/mL, L3482, Invitrogen) in presence or not of the fluorescent conjugate (Fc(A680)-VH4127 or Fc(A680)-VH4Sc, 20 nM) in DMEM-1% BSA for 6 h. When the lysotracker blue DND-22 (1/10,000, L7525, Invitrogen) was added one hour before the end of incubation, nuclei were stained with Hoechst (10 µg/mL, Invitrogen) and an adhesive silicone isolator (Sigma-Aldrich) was applied on glass coverslips for live-cell microscopy analysis. In absence of lysotracker blue DND-22 addition, cells were fixed in 4% paraformaldehyde (PFA), nuclei were stained as above and glass coverslips were mounted with Prolong Glass Antifade Mountant (Invitrogen).

### Confocal microscopy imaging

Cell and tissue fluorescent images were acquired with a laser-scanning confocal microscope (LSM700, Zeiss) using Plan Apochromat ×63 oil immersion objective lens and were analyzed with Zen Lite 2012 software.

### FACS analysis of cell surface and internalized fluorescent LDL and conjugates

7000 *Ldlr* WT or KO PK4A cells were grown in DMEM-10% FBS for 48 h. Then, cells were incubated with Fc(A680)-VH4127 or Fc(A680)-VH4Sc (20 nM) in presence or not of DiI-LDL (20 µg/ml) in DMEM-1% BSA for 6 h. Half of the cells were additionally treated with an acid stripping solution (0.15 M NaCl, 0.2 M glycine, pH 3) to remove the cell-surface LDL and/or conjugate. Finally, all cells were fixed in 2% PFA and 5 mM EDTA before to be analyzed by FACS (Attune NxT flow cytometer, ThermoFisher) using the Attune NxT Software version 3.1.

### Preclinical mouse models

Management of mouse KI/KIC breeding colony and experimental mouse procedures were both authorized by the local ethics committee for animal experimentation (CEEA14, Marseille, France) (Ethics approval numbers: 01527-02, 9749-2017042710417337 and 24209-2020021719581546).

#### Orthotopic PDAC xenograft mouse models

Local analgesic and anesthetic agents (buprenorphine (0.2 mg/kg) and lidocaine (3.5 mg/kg), respectively) were administered to 5-weeks old female athymic mice (Hsd:Athymic Nude-Foxn1nu, *Mus musculus*, Envigo, France). Then, BxPC-3, MIA PaCa-2 or PANC-1 cells (1.5 × 10^6^ cells) were implanted in the pancreas under 1.5% isoflurane anesthesia delivered in oxygen-supplemented air (flow rate of 0.7 and 0.3 L/min, respectively) and stable core temperature. Mice were sacrificed 3-, 4- or 8-weeks after BxPC-3, MIA PaCa-2, or PANC-1 cell implantation. Half of the pancreatic tumor samples were fixed in 10% (wt/vol) formalin, while the other half were fast-frozen in cold isopentane for further analysis.

#### Subcutaneous PDAC mice

Five-week-old female athymic mice (Hsd:Athymic Nude-Foxn1^nu^, *Mus musculus*, Envigo, France) were implanted subcutaneously between the shoulder blades with 2 × 10^6^ *Ldlr* WT or KO PK4A cells. Implantation was performed under anesthesia, as described above. Imaging experiments were performed when the calculated tumor volume ((π/6) × [(Length × Width)/2]^3^)^43^ reached 300 ± 21 mm^3^.

#### Spontaneous PDAC mice

We euthanized male and female KIC mice (*Mus musculus, LSL-Kras*^*G12D*^*; Ink4a/Arf*^*fl/fl*^*; Pdx1-Cre*)^11^ when severe PDAC symptoms appeared (≈9-weeks old), and their age-matched control mice (KI mice: *LSL-Kras*^*G12D*^*; Ink4a/Arf*^*fl/fl*^, *Mus musculus*).

#### Induced-chronic pancreatitis mice

Seven-week-old male and female KI mice (*Mus musculus*) were daily injected with cӕrulein (250 µg/kg, Sigma-Aldrich) for 12 days by intraperitoneal route.

#### Intra-hepatic PDAC mice

Seven-week-old male and female KI mice (*Mus musculus*) were implanted with 1.5 × 10^5^ PK4A cells in two different liver lobes. Mice were previously injected with local analgesic and anesthetic agents and surgery was performed under anesthesia as described above. Imaging experiments were started 15 days after implantation.

### In vivo imaging and tissue distribution of fluorescent conjugates

A whole-body fluorescence acquisition was performed before IV injection (tail or retro-orbital sinus) of 1 nmole of conjugate (Fc(A680)-VH4127 or Fc(A680)-VH4Sc) and at different post-injection times using the PhotonIMAGER and the PhotoAcquisition software (Biospace Lab, France). For KI and KIC mice, hair of the abdominal part was removed to increase fluorescence detection. At the time of imaging, a visible light image followed by an Alexa Fluor 680 acquisition (Ex.: 660 nm, Em. 700 nm, acquisition time: 2 s) were realized on anesthetized mice. Then, mice were euthanized and fluorescence acquisitions of whole-body after laparotomy and/or of excised organs of interest were performed. Finally, half of the tissue samples were fixed in 10% (wt/vol) formalin, while the other half were fast-frozen in cold isopentane for further analysis. Analysis of fluorescence specific to a region of interest (ROI) was performed using M3 Vision software (version 1.1.2.26170, Biospace Lab, France). For whole-body acquisitions, the ROI autofluorescence, measured before injection, was subtracted from the ROI fluorescence specific to each conjugate measured at each post-injection time.

### Measurements of key serum indicators of hepato- and nephrotoxicity

Retro-orbital blood samples were collected under anesthesia 24 h and 48 h after injection of Fc(A680)-VH4127 (1 nmole/mouse) or vehicle (NaCl 0.9%, equivalent volume) in KI and KIC mice. Serum was separated from red blood cells by centrifugation (3000 rpm, 20 min, 4 °C) and either directly used for aspartate aminotransferase (AST) activity assay (MAK055, Sigma-Aldrich) or previously deproteinized using 10 kDa MWCO spin filter (Vivacon 500, Sartorius) for creatinine content measurments (MAK080, Sigma-Aldrich) according to the manufacturer’s instructions.

### Statistics and reproducibility

Two-tailed unpaired Student *t*-test or one-way/two-way ANOVA followed by post-hoc Tukey honest significant difference (HSD) test was used to respectively highlight significant differences between two or more than two experimental groups (GraphPad Prism 6.01 software). A *p*-value < 0.05 was used as the cut-off for significance and levels of significance were denoted as *p < 0.05, **p < 0.01, and ***p < 0.001. Number of human samples, animals or independent experiments (*n*) are indicated in the figure legend. Data, expressed as mean ± standard error of mean (s.e.m.), show high reproducibility between replicate experiments.

### Reporting summary

Further information on research design is available in the Nature Research Reporting Summary linked to this article.

## Supplementary information

Supplementary Information

Description of Supplementary Files

Supplementary Data 1

Reporting Summary

## Data Availability

All mRNA expression datasets from human pancreatic samples used in this study are listed in Supplementary Table S1 and unprocessed blots and membrane stained with amido black are provided as Supplementary Fig. 8 in the Supplementary Information file. Moreover, source data underlying all the main and supplemental graphs and tables illustrated in this manuscript are available in the Supplementary Data file.
