# Supplementary material for: LDL receptor-peptide conjugate as in vivo tool for specific targeting of pancreatic ductal adenocarcinoma
Source: Commun Biol. 2021 Aug 19;4:987. doi: 10.1038/s42003-021-02508-0 (PMC8377056; doi:10.1038/s42003-021-02508-0)
Supplement: Supplementary file 1 — Supplementary Information [file 42003_2021_2508_MOESM1_ESM.pdf]

LDL receptor-peptide conjugate as *in vivo* tool for specific targeting of pancreatic ductal adenocarcinoma

Angélina Acier[1,2], Magali Godard[2#], Fanny Gassiot[2#], Pascal Finetti[1], Marion Rubis[1], Jonathan Nowak[2], François Bertucci[1], Juan L. Iovanna[1], Richard Tomasini[1], Pascaline Lécorché[2], Guillaume Jacquot[2], Michel Khrestchatisky[3], Jamal Temsamani[2], Cédric Malicet[2], Sophie Vasseur[1‡] and Fabienne Guillaumond[1‡*]

Supplementary methods

Immunostaining on formalin-fixed paraffin-embedded (FFPE) tissue sections

FFPE sections were deparaffinised before to be incubated in boiled target retrieval buffer solution (TRS pH 6, Dako) and then with inhibitor of endogenous peroxidase activity.

For co-immunofluorescence staining, BxPC-3 tumor xenograft slices were first co-incubated with goat anti-human LDLR antibody (1/100, AF2148, R&D Systems) and rabbit anti-human pan-Cytokeratin (1/50, ab9377, Abcam) and then with Alexa Fluor 488 donkey anti-goat IgG (1/500, A11055, Invitrogen) and Alexa Fluor 568 goat anti-rabbit IgG (1/500, A11036, Invitrogen).

For immunohistochemistry (IHC), tissue sections were incubated with blocking solution and then incubated with goat anti-mouse LDLR (1/150, AF2255, R&D Systems) or goat anti-human LDLR antibody (1/100). LDLR staining was revealed using the high-sensitivity avidin-biotin based detection kit (PK-6105, Vector Laboratories) and liquid-DAB$^+$ substrate chromogen system (Dako). Finally, sections were counterstained with Mayer's hematoxylin solution, and then incubated with bluing reagent (0.1% sodium bicarbonate) and mounted with coverslips.

Immunostaining on frozen PDAC sections

10 µm cryostat sections of PDAC embedded in optimal cutting temperature (OCT) compound were fixed in 4% paraformaldehyde (PFA) and incubated in a permeabilization and blocking solution (0.1% Triton X-100, 3% bovine serum albumin (BSA)). Then, tissue sections were first incubated with goat anti-mouse LDLR (1/100, AF2255, R&D Systems) and Alexa Fluor 594 goat anti-human IgG, Fcγ fragment specific (1/200, 109-585-098,

Jackson ImmunoResearch) antibodies, and then with Alexa Fluor 488 donkey anti-goat IgG antibody (1/500, A11055, Invitrogen). Finally, a nuclei staining with DAPI (1 µg/mL, ThermoScientific) was performed on PDAC sections before to be mounted with Prolong Glass Antifade Mountant (Invitrogen).

LDLR immunofluorescence in 2D and 3D murine PDAC cells

Ldlr WT or KO PK4A organoids were fixed in 4% PFA, coated in histogel (HG-4000-012, Thermo Scientific) and embedded in paraffin. In 2D, 10,000 cells, grown on glass coverslips for 48h, were fixed in 4% PFA and permeabilized or not with 0.1% Triton X-100 in PBS 1X. Then, cells and organoids sections were sequentially incubated in a 3% BSA blocking solution, with goat anti-mouse LDLR antibody (1/100, AF2255, R&D Systems) and with Alexa Fluor 488 donkey anti-goat IgG antibody (1/500, A11055, Invitrogen). Finally, a nuclei staining with DAPI (1 µg/mL, ThermoScientific) was performed on PDAC sections before to be mounted with Prolong Glass Antifade Mountant (Invitrogen).

Haematoxylin phloxine/eosin saffron coloration (HPS/HES)

After deparaffinization, tissue sections were stained with haematoxylin (HX8771749, Merck Millipore) and differentiate with 1% acid alcohol (1% hydrochloric acid in 96% alcohol). After an incubation in bluing reagent solution (0.5% sodium bicarbonate), tissue sections were stained with 2.5% phloxine solution (361470-0025, RAL Diagnostic) and then with saffron (369200-0500, RAL Diagnostic).

The HES coloration was processed using the Discovery XT Automated slide staining system (Ventana Medical System, USA) and the following dyes: haematoxylin (CS70030, Dako), eosin (CS70130, Dako) and saffron (4 g/L, F/SAFRAN, Spigol). Finally, tissue sections were dehydrated and mounted with coverslips.

Total protein extracts

Murine tissues were grounded with ceramic- or stainless-steel beads in freshly prepared RIPA 1X buffer using the Precellys homogenizer (Bertin Technologies). RIPA 1X buffer was composed of 10% Na-deoxycholate, 0.1% SDS, 1% Triton X-100, 10 mM Tris pH 8, 140 mM NaCl, and supplemented with protease (1/200, P8340, Sigma-Aldrich) and phosphatase inhibitors (1% phenylmethylsulfonyl fluoride, 1 mM sodium fluoride, 100 µM sodium orthovanadate and 40 mM β-glycerophosphate). Tissue homogenates were centrifuged (14,000 rpm, 10 min, 4°C), and supernatants corresponding to total protein extracts were collected and stored at -80°C until use.

Cells were lysed in 50 mM Tris pH 8, 150 mM NaCl, 1% NP-40, supplemented with protease and phosphatase inhibitors, as described above. After 20 min of incubation at 4°C under agitation, cell lysates were centrifuged (14,000 rpm, 10 min, 4°C) and total protein extracts were collected and stored at -80°C until use.

Western-blot

Sample protein concentration was determined using the Bio-Rad protein (λ = 595 nm). After denaturation, 50 µg of proteins were separated by sodium dodecyl sulfate polyacrylamide gel electrophoresis (SDS/PAGE) on a 10% acrylamide bis-acrylamide gel

(29:1, Euromedex) and then transferred onto 0.2 µm nitrocellulose membranes (Bio-Rad). Membranes were blocked in 5% (wt/vol) non-fat milk in TBS 1X (50 mM Tris-HCl, 150 mM NaCl, pH 7.5) before an overnight incubation at 4°C with goat anti-mouse LDLR (1/1000, AF2255, R&D Systems), goat anti-human LDLR (1/1000, AF2148, R&D Systems) or mouse anti-β-tubulin antibody (1/10000, T4026, Sigma-Aldrich). After repeated washing in TBS 1X-0.1% Tween-20, membranes were incubated (1h, room temperature) with appropriate HRP secondary antibody (1/3000 for rabbit anti-goat or 1/5000 for goat anti-mouse, 6160-05 or 1030-05, Southern Biotech). Finally, LDLR and β-tubulin were detected using Immobilon Western Chemiluminescent HRP Substrate (Milipore) and the PXi chemiluminescent imager (Syngene). Relative LDLR quantity was determined by densitometry using ImageJ software (NIH) and normalized to relative β-tubulin or total transferred proteins stained with amido black solution (0.1% naphtol blue black, 10% methanol, 2% acetic acid).

Supplementary figures

Supplementary Fig. 1: LDLR is a promising cell-surface receptor for targeting human PDAC.

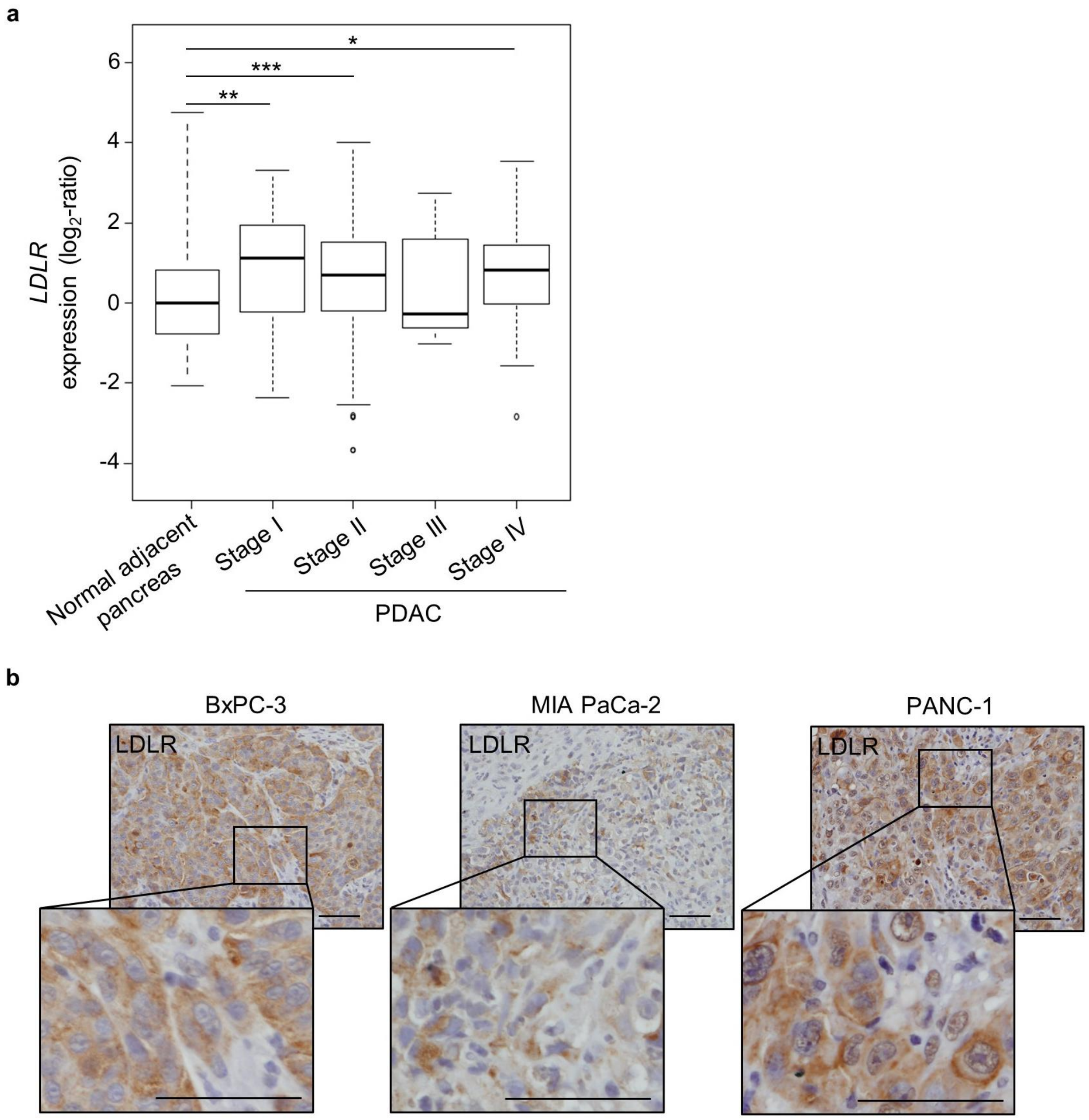

(**a**) Box-and-whisker plots representing the median value of human *LDLR* expression in primary PDAC, according to American Joint Committee on Cancer stage, as compared to the median value of LDLR expression across normal adjacent pancreas samples and

expressed as log_2-ratio (stage I: n=59, stage II: n=422, stage III: n=10 and stage IV: n=41 biologically independent samples). Significant differences in *LDLR* expression between each human tissue samples (PDAC at different stages and normal adjacent pancreas) are indicated. One-way ANOVA with post-hoc Tukey HSD test, *: $p < 0.05$, **: $p < 0.01$, ***: $p < 0.001$. (**b**) Representative LDLR IHC images of BxPC-3, MiaPaCa-2 and PANC-1 orthotopic xenograft sections. 10x magnification, scale bar: 100 µm. An enlarged image of indicated part is provided in inset box. n=3 mice/xenograft type.

Supplementary Fig. 2: Total loss of LDLR in *Ldlr* knock-out (KO) PK4A cells generated by genome editing.

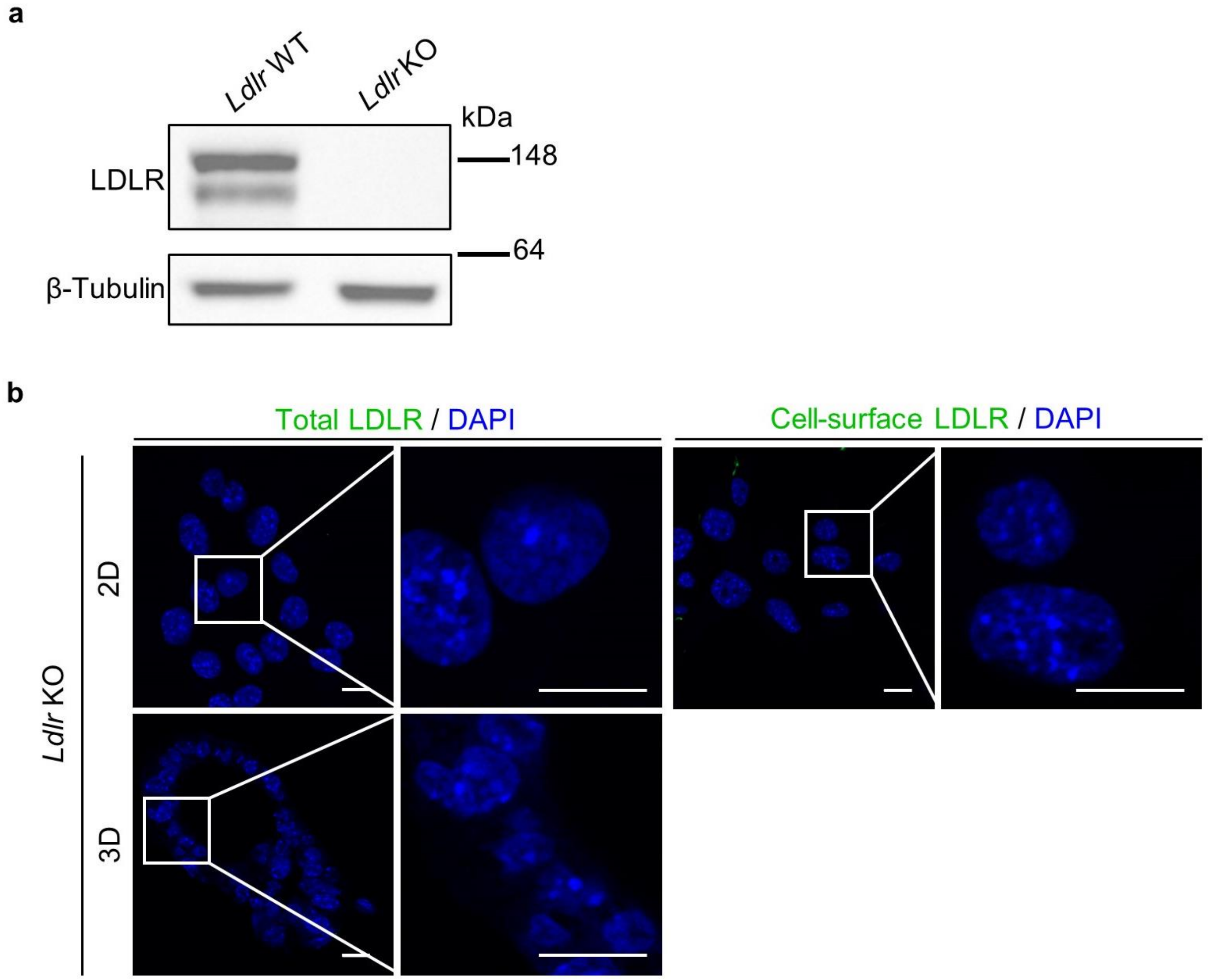

(**a**) Representative western-blot of LDLR protein levels in *Ldlr* wild-type (WT) and *Ldlr* KO PK4A cells. β-Tubulin is shown as a loading control. n=5 independent experiments. (**b**) Representative confocal microscopy images showing the absence of total (*i.e.* bound and internalized) and/or cell-surface LDLR (in green color) in 2D and 3D *Ldlr* KO cells. 60x magnification, scale bar: 10 µm. An enlarged merge image of indicated part is provided in inset box. n=2 independent experiments.

Supplementary Fig. 3: Absence of control conjugate internalization and intracellular trafficking in *Ldlr* KO PK4A cells.

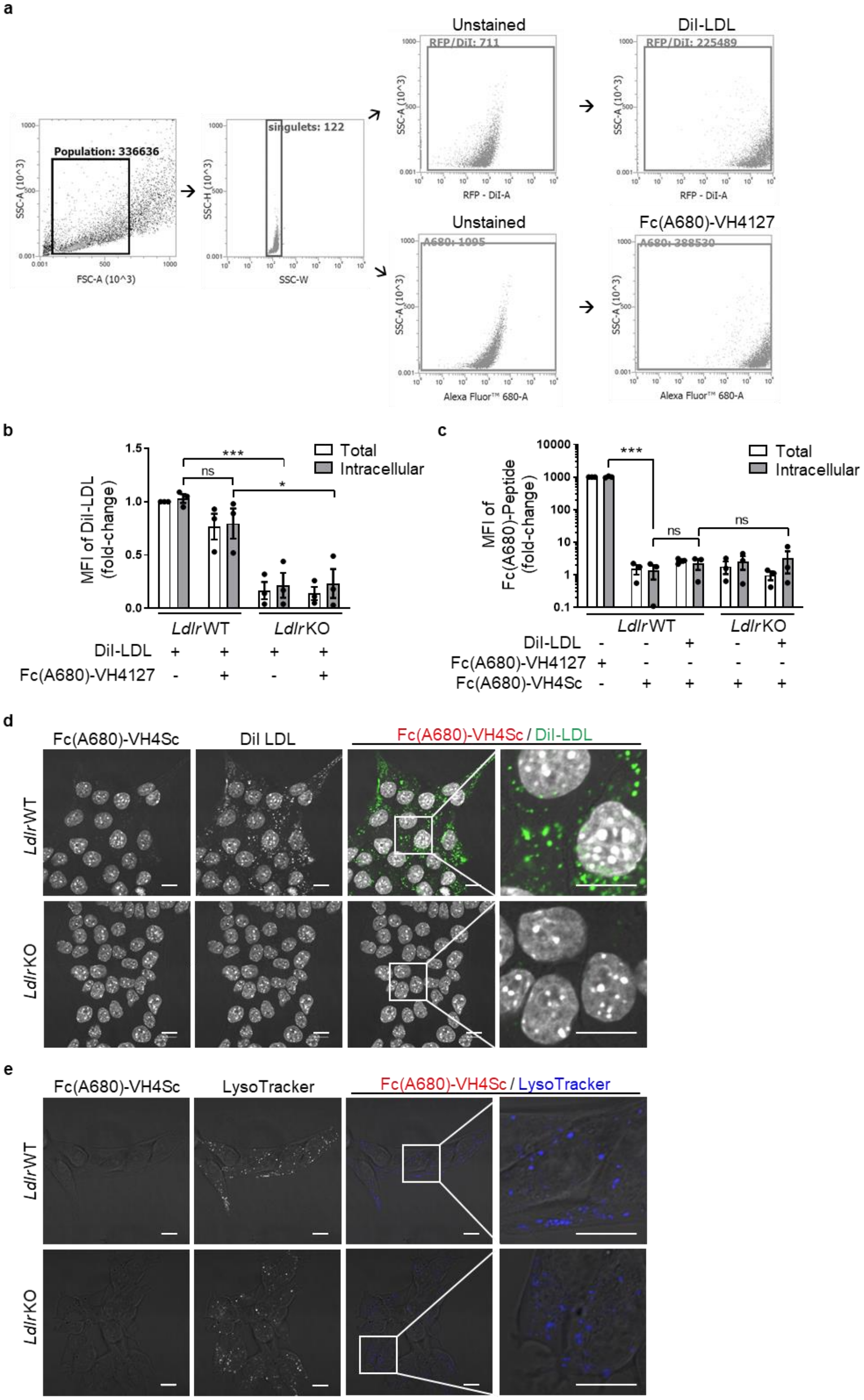

(**a**) Flow cytometry gating procedure for PK4A cell analysis. A loose gate containing cell population of interest and excluding debris was draw in SSC-A x FSC-A plot. Then, single cells from previous gated cell population were exclusively selected from SSC-H x SSC-W plot. Finally, positive DiI-LDL or Alexa Fluor 680 cells were discriminated from unstained cells. (**b**) FACS analysis of total and internalized DiI-LDL in *Ldlr* WT and KO PK4A cells incubated with or without Fc(A680)-VH4127. Data are represented as fold-change ± s.e.m. of the mean of fluorescence intensity (MFI) of DiI-LDL expressed relative to MFI measured in *Ldlr* WT cells without conjugate and arbitrarily set to 1. (**c**) FACS analysis of total- and internalized Fc(A680)-VH4Sc, with or without DiI-LDL, in *Ldlr* WT and KO PK4A cells. Data are represented as fold-change of MFI of the conjugate ± s.e.m. and expressed relative to total Fc(A680)-VH4127 in *Ldlr* WT arbitrarily set to 1000. (**b-c**) One-way ANOVA with post-hoc Tukey HSD test, ns: no significant difference, *: $p < 0.05$, ***: $p < 0.001$. n=3 independent experiments. (**d**) Representative confocal microscopy images of Fc(A680)-VH4Sc (pseudo-coloured red) and DiI-LDL (pseudo-coloured green) in fixed *Ldlr* WT and KO PK4A cells. (**e**) Representative confocal microscopy images of Fc(A680)-VH4Sc (pseudo-coloured red) and LysoTracker blue in live *Ldlr* WT and KO PK4A cells. (**c-d**) 60x magnification, scale bar: 10 µm. An enlarged merge image of indicated part is provided in inset box. n=3 independent experiments.

Supplementary Fig. 4: Absence of subcutaneous PDAC targeting with the control conjugate.

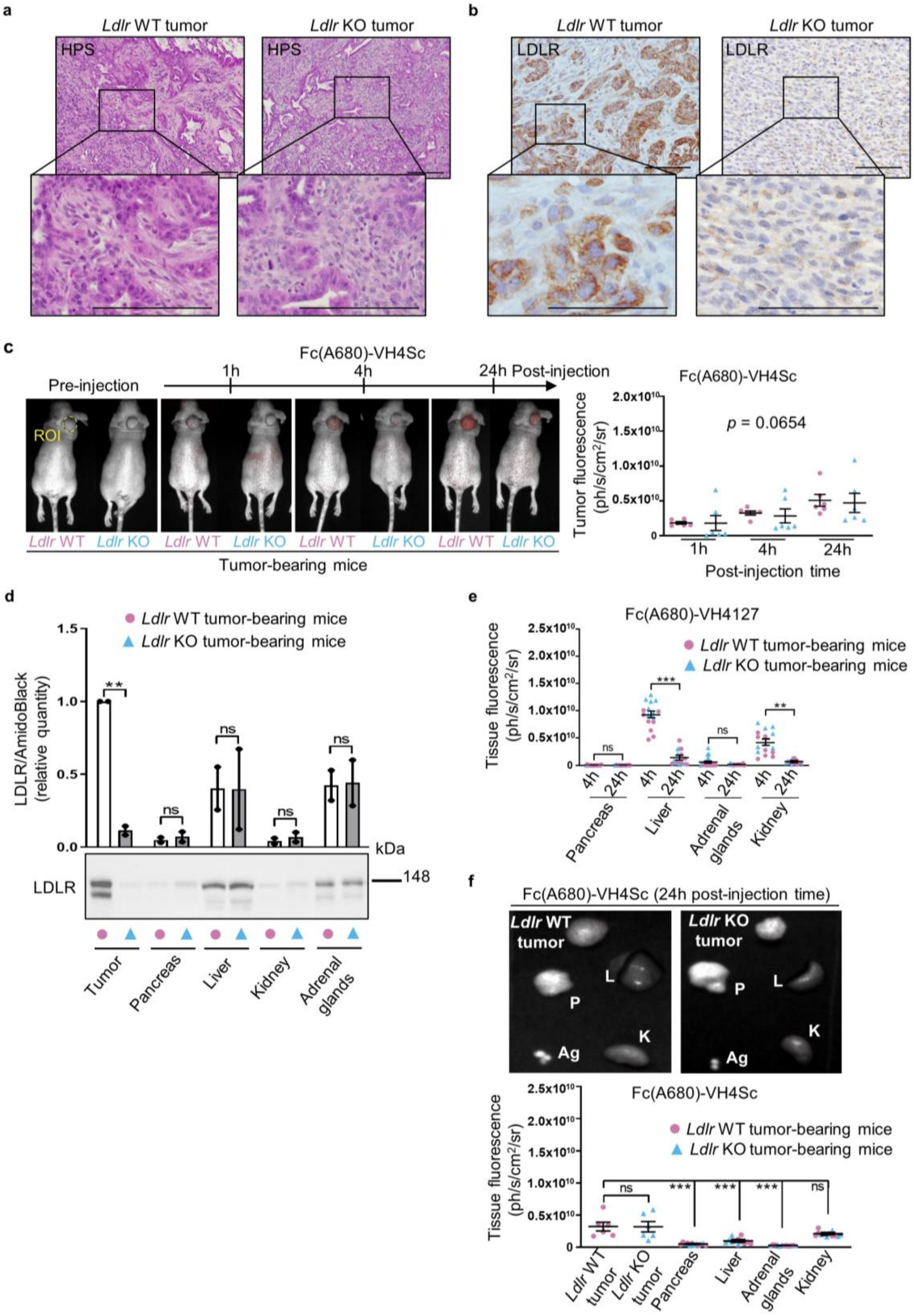

Representative images of HPS (**a**) (10x magnification, scale bar: 500 µm) or LDLR staining (**b**) (20x magnification, scale bar: 100 µm) in subcutaneous *Ldlr* WT and KO tumors. An enlarged image of indicated part is provided in inset box. n=5 mice/group. (**c**) Representative images and fluorescence quantitation of *Ldlr* WT and KO tumor-bearing mice before and 1, 4 and 24h after Fc(A680)-VH4Sc injection (1 nmole/mouse). n=6 mice/group. (**d**) Representative LDLR immunoblot and LDLR quantitation in tumors and healthy tissues from *Ldlr* WT and KO tumor-bearing mice. LDLR/amido black ratios, represented as mean ± s.e.m., are expressed relative to *Ldlr* WT tumor ratio arbitrarily set to 1. n=2 independent experiments. (**e**) Fc(A680)-VH4127 fluorescence at 4 and 24h post-injection in healthy organs from *Ldlr* WT and KO tumor-bearing mice. n=8 or 9 mice/time/group. (**f**) Representative fluorescence image of *Ldlr* WT and KO tumors, pancreas (P), liver (L), adrenal glands (Ag) and kidney (K) 24h after Fc(A680)-VH4Sc administration. (**c, e-f**) Conjugate fluorescence in organ/tumor area is expressed as mean (ph/s/cm^2/sr) ± s.e.m. One-way ANOVA with post-hoc Tukey HSD test, ns: no significant difference, **: $p < 0.01$, ***: $p < 0.001$.

Supplementary Fig. 5: High LDLR levels in spontaneous PDAC.

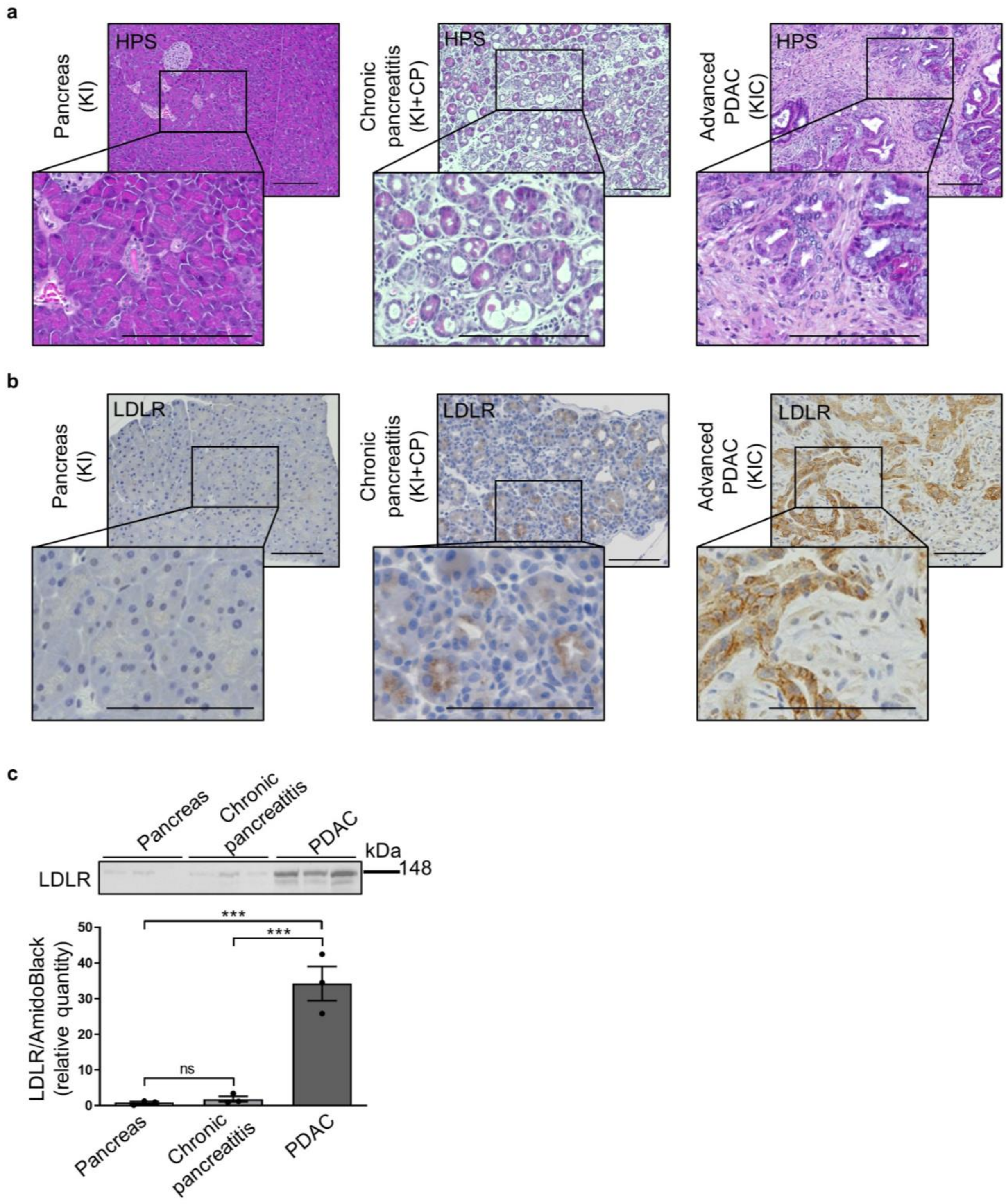

(**a-b**) Representative images of Haematoxylin Phloxine Saffron (HPS) (**a**) or LDLR staining (**b**) in healthy, inflammatory and tumoral pancreas from 9-weeks old KI, KI+CP

and KIC mice, respectively. (**a**) 10x magnification, scale bar: 500 µm and (**b**) 20x magnification, scale bar: 100 µm. (**a-b**) An enlarged image of indicated part is provided in inset box. n=3 mice/group. (**c**) Immunoblot of LDLR proteins in healthy, inflammatory and tumoral pancreas from KI, KI+CP and KIC mice, respectively. Proteins transferred onto membranes and revealed by amido black staining are used as a loading control. Quantitation of the LDLR/transferred proteins ratio in each tissue is shown. Data are represented as mean ± s.e.m. and expressed relative to ratio obtained in healthy pancreas and arbitrarily set to 1. One-way ANOVA with post-hoc Tukey HSD test, ns: no significant difference, ***: $p < 0.001$. n=3 mice/group.

Supplementary Fig. 6: Tissue LDLR profile in KI, KI+CP and KIC mice and distribution of Fc(A680)-VH4127 in healthy tissues 24h and 48h after its injection in KI and KIC mice.

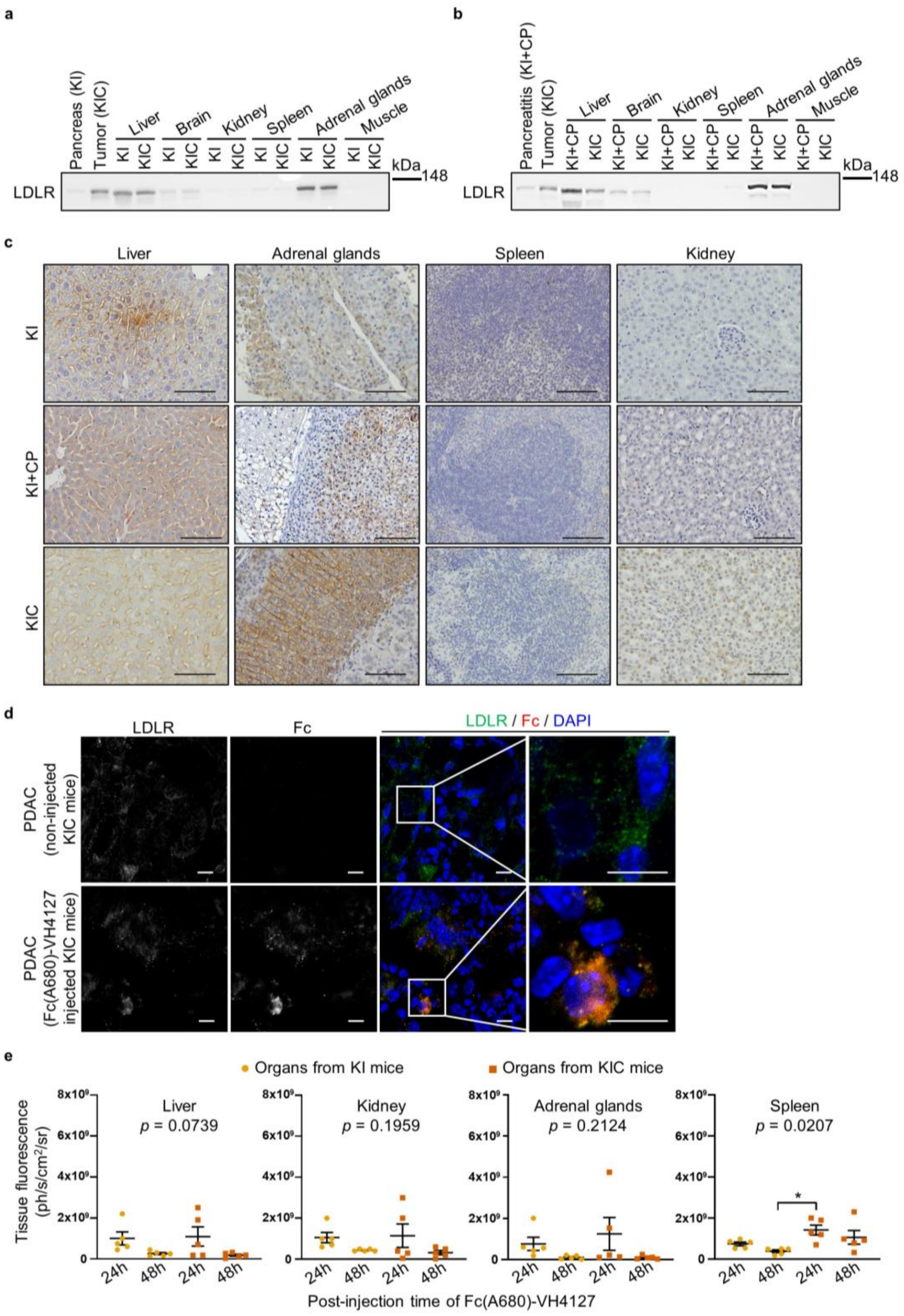

(**a**) Representative immunoblot of LDLR proteins in PDAC (KIC mice) as compared to healthy tissues from KI and KIC mice. (**b**) Representative immunoblot of LDLR proteins in PDAC (KIC mice) as compared to inflammatory pancreas (KI+CP) and other healthy tissues from KIC and KI+CP mice. (**a-b**) Proteins transferred onto membranes and revealed by amido black staining are used as a loading control. n=2 independent experiments. (**c**) Representative images of LDLR staining in the indicated healthy tissues from KI, KI+CP and KIC mice. 20x magnification, scale bar: 100 µm. n=3 mice/group. (**d**) Representative confocal microscopy images of PDAC sections from KIC mice injected or not with Fc(A680)-VH4127 and stained with anti-mouse LDLR (in green color) and anti-human IgG1 Fc fragment antibody (in red color). 60x magnification, scale bar: 10 µm. An enlarged merge image of indicated part is provided in inset box. n=3 mice/group. (**e**) Quantification of Fc(A680)-VH4127 fluorescence at 24 and 48h post-injection in the liver, kidney, adrenal glands and spleen of KI and KIC mice. Data are expressed as mean (ph/s/cm^2/sr) ± s.e.m. One-way ANOVA with post-hoc Tukey HSD test. n=5 KI and KIC mice.

Supplementary Fig. 7: Absence of liver and kidney damages caused by the injection of Fc(A680)-VH4127 conjugate in KI and KIC mice.

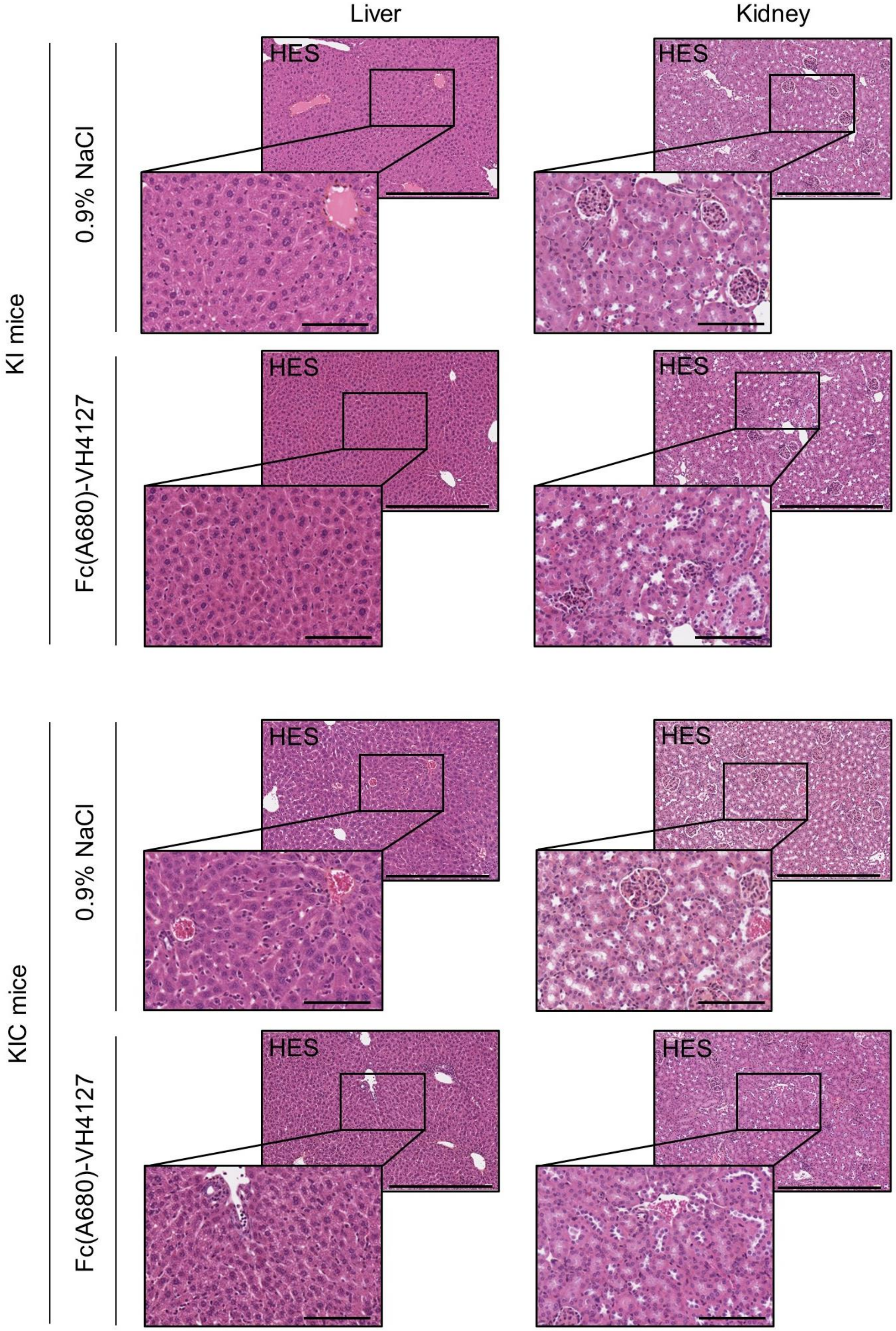

Representative images of Haematoxylin Eosin Saffron (HES) coloration of liver and kidney sections from KI or KIC mice injected with the Fc(A680)-VH4127 (1 nmole/mouse) conjugate or vehicle (0.9% NaCl, equivalent volume). 10x magnification, scale bar: 500 µm. An enlarged image of indicated part is provided in inset box, scale bar: 100 µm. n=3 mice/group.

Supplementary Fig. 8: Unprocessed blots and membrane stained with amido black.

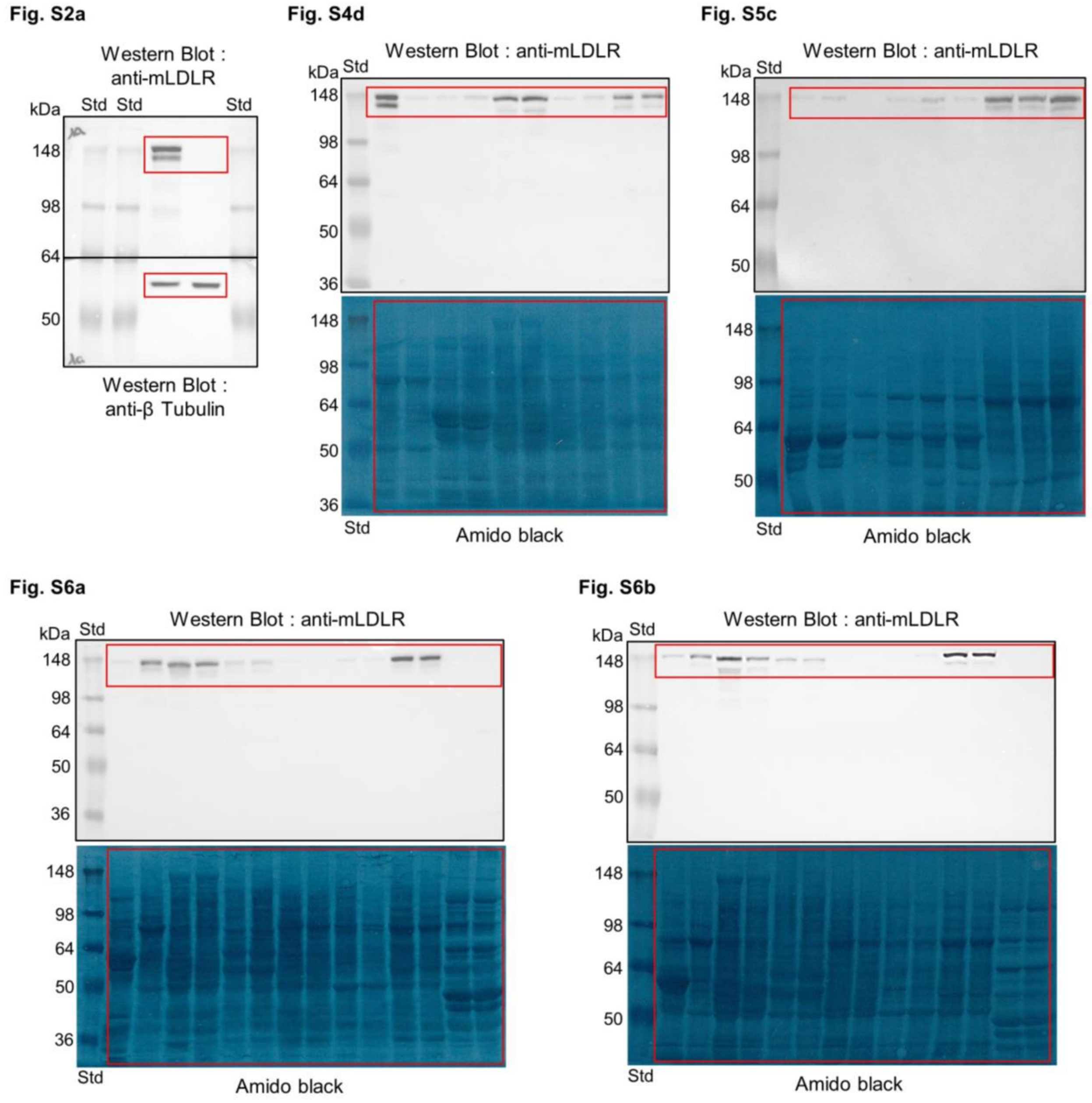

Supplementary tables

Supplementary Table S1: Publicly available pancreatic cancer genomic datasets used for meta-analysis of human *LDLR* gene expression.

References	Data sources	Technological platforms	Probes number	NAP	PDAC	Meta-stasis	Total samples included
Badea L. et al., 2008	GEO database, GSE15471	Affymetrix, array U133 Plus 2.0	54K	39	36	0	75 (8.3%)
Bailey P. et al., 2016	EGA database, EGAS00001000154	Illumina, RNA sequencing HiSeq	18K	0	96	0	96 (10.7%)
Chen D.T. et al., 2015	GEO database, GSE57495	Affymetrix, Rosetta/Merck RSTA Custom 2.0	60K	0	63	0	63 (7%)
Collisson E.A. et al., 2011	GEO database, GSE17891	Affymetrix, array U133 Plus 2.0	54K	0	27	0	27 (3%)
Grutzmann R. et al., 2004	Array-Express database, E-MEXP-950	Affymetrix, array U133 A+B	22K+ 22K	0	11	3	14 (1.6%)
Kirby M.K. et al., 2016	GEO database, GSE79670	Illumina, RNA sequencing HiSeq	49K	0	51	0	51 (5.7%)
Lunardi S. et al., 2014	GEO database, GSE55643	Agilent, array 4x44K G4112F (014850)	44K	8	45	0	53 (5.9%)
Moffitt R.A. et al., 2015	GEO database, GSE71729	Agilent, array 4x44K G4112F (014850)	44K	0	0	61	61 (6.8%)
Monzon F.A. et al., 2009	GEO database, GSE12630	Affymetrix, array U133 A	22K	0	24	0	24 (2.7%)
Park M. et al., 2014	GEO database, GSE43795	Illumina, array Human HT-12 V4.0	48K	0	6	0	6 (0.7%)
Straford J.K. et al., 2010	GEO database, GSE21501	Agilent, array 4x44K G4112F (014850)	44K	0	132	0	132 (14.7%)
TCGA, PAAD	TCGA portal, https://tcga-data.nci.nih.gov	Illumina, RNA sequencing V2	25K	4	150	1	155 (17.2%)
van den Broeck A. et al., 2012	GEO database, GSE42952	Affymetrix, array U133 Plus 2.0	54K	0	12	11	23 (2.6%)
Winter C. et al., 2012	Array-Express database, E-MEXP-2780	Affymetrix, array U133 Plus 2.0	54K	0	30	0	30 (3.3%)
Zhang G. et al., 2013	GEO database, GSE28735	Affymetrix, array Gene 1.0 ST	33K	45	45	0	90 (10%)
Total				96	728	76	900 (100%)

NAP: Normal Adjacent Pancreas, PDAC: Pancreatic Ductal Adenocarcinoma, PAAD: Pancreatic Adenocarcinoma, GEO: Gene Expression Omnibus, EGA: European Genome-phenome Archive, TCGA: The Cancer Genome Atlas.

Supplementary Table S2: Sequence and kinetic parameters of the interaction between VH4127 peptide and murine (m) or human (h) LDLR.

		VH4127 [a]Pr-[cMThzRLRGPen]c-NH_2		
		k_{on} (1/Ms)	k_{off} (1/s)	K_D (M)
mLDLR	Mean	1.66×10^{6}	8.21×10^{-2}	5.10×10^{-8}
	SD	4.48×10^{5}	1.57×10^{-3}	1.39×10^{-8}
hLDLR	Mean	4.06×10^{6}	8.94×10^{-2}	2.16×10^{-8}
	SD	1.74×10^{6}	4.47×10^{-2}	1.60×10^{-9}

[a]Natural amino acids in the VH4127 sequence are indicated by one letter from amino acid code. The non-natural amino acids, Thz (thiazolidine-4-carboxylic acid) and Pen (penicillamin) have replaced the proline and cysteine residues, respectively. (D)-configuration is indicated by "c" and "Pr-" means N-terminal propionylation. The association rate constant (k_{on}), the dissociation rate constant (k_{off}) and the dissociation constant (K_D) of VH4127 to LDLR are indicated as mean ± standard deviation (SD).
