# Supplementary material for: LDL receptor-peptide conjugate as in vivo tool for specific targeting of pancreatic ductal adenocarcinoma
Source: Commun Biol. 2021 Aug 19;4:987. doi: 10.1038/s42003-021-02508-0 (PMC8377056; doi:10.1038/s42003-021-02508-0)
Supplement: Supplementary file 2 — Description of Supplementary Files [file 42003_2021_2508_MOESM2_ESM.pdf]

Description of Additional Supplementary Files

File name: Supplementary Data 1

Description:

Sheet 1: Table of Contents

Sheet 2: Source data for human LDLR expression (Figures 1a-1b, Supplementary Figure S1a)

Sheet 3: Source data for human LDLR staining (Figures 1c-1d)

Sheet 4: Source data for Fc(A680)-VH4127 conjugate and VH4127 peptide kinetic properties (Figure 3b, Supplementary Table S2)

Sheet 5: Source data for flow cytometry (Figures 2b-3c, Supplementary Figures S3b-S3c)

Sheet 6: Source data for mouse LDLR western blot (Supplementary Figures S4d-S5c)

Sheet 7: Source data for subcutaneous tumor imaging (Figures 4a-4b, Supplementary Figures S4c-S4e-S4f)

Sheet 8: Source data for spontaneous tumor imaging (Figures 5a-5b-5c, Supplementary Figure S6e)

Sheet 9: Source data for serum AST and creatinine assays (Figure 5d)

Sheet 10: Source data for liver metastasis imaging (Figure 6e)
