# Supplementary material for: LDL receptor-peptide conjugate as in vivo tool for specific targeting of pancreatic ductal adenocarcinoma
Source: Commun Biol. 2021 Aug 19;4:987. doi: 10.1038/s42003-021-02508-0 (PMC8377056; doi:10.1038/s42003-021-02508-0)
Supplement: Supplementary file 4 — Reporting Summary [file 42003_2021_2508_MOESM4_ESM.pdf]

nature research

Corresponding author(s): Fabienne GUILLAUMOND

Last updated by author(s): Jul 19, 2021

# Reporting Summary

Nature Research wishes to improve the reproducibility of the work that we publish. This form provides structure for consistency and transparency in reporting. For further information on Nature Research policies, see our Editorial Policies and the Editorial Policy Checklist.

## Statistics

For all statistical analyses, confirm that the following items are present in the figure legend, table legend, main text, or Methods section.

| n/a | Confirmed | |
|---|---|---|
| ☐ | ☒ | The exact sample size (*n*) for each experimental group/condition, given as a discrete number and unit of measurement |
| ☐ | ☒ | A statement on whether measurements were taken from distinct samples or whether the same sample was measured repeatedly |
| ☐ | ☒ | The statistical test(s) used AND whether they are one- or two-sided<br>*Only common tests should be described solely by name; describe more complex techniques in the Methods section.* |
| ☐ | ☒ | A description of all covariates tested |
| ☐ | ☒ | A description of any assumptions or corrections, such as tests of normality and adjustment for multiple comparisons |
| ☐ | ☒ | A full description of the statistical parameters including central tendency (e.g. means) or other basic estimates (e.g. regression coefficient) AND variation (e.g. standard deviation) or associated estimates of uncertainty (e.g. confidence intervals) |
| ☐ | ☒ | For null hypothesis testing, the test statistic (e.g. *F*, *t*, *r*) with confidence intervals, effect sizes, degrees of freedom and *P* value noted<br>*Give P values as exact values whenever suitable.* |
| ☒ | ☐ | For Bayesian analysis, information on the choice of priors and Markov chain Monte Carlo settings |
| ☒ | ☐ | For hierarchical and complex designs, identification of the appropriate level for tests and full reporting of outcomes |
| ☒ | ☐ | Estimates of effect sizes (e.g. Cohen's *d*, Pearson's *r*), indicating how they were calculated |

*Our web collection on statistics for biologists contains articles on many of the points above.*

## Software and code

Policy information about availability of computer code

| | |
|---|---|
| Data collection | Data were collected from the National Center for Biotechnology Information (NCBI)/Genbank GEO, ArrayExpress, and TCGA databases (full references are provided in supplementary table 1. |
| Data analysis | Individual Affymetrix dataset was normalized using robust multichip average with the non-parametric quantile algorithm for the raw Affymetrix data (Bioconductor and associated-packages, R software), while non-Affymetrix dataset (i.e. Agilent, Illumina) was normalized with quantile normalisation procedure. Then, Agilent or Affymetrix hybridization probes were mapped across different technological platforms (Stanford Online Universal Resource for Clones and ESTs (SOURCE), NCBI EntrezGene and Affymetrix NetAffx Annotation files). |

For manuscripts utilizing custom algorithms or software that are central to the research but not yet described in published literature, software must be made available to editors and reviewers. We strongly encourage code deposition in a community repository (e.g. GitHub). See the Nature Research guidelines for submitting code & software for further information.

## Data

Policy information about availability of data

All manuscripts must include a data availability statement. This statement should provide the following information, where applicable:

- Accession codes, unique identifiers, or web links for publicly available datasets
- A list of figures that have associated raw data
- A description of any restrictions on data availability

The authors declare that all data generated from publicly available datasets and supporting the findings of this study are available within the supplementary table S1. The Raw data associated with main and supplementary figures are provided in Supplementary data file.

# Field-specific reporting

Please select the one below that is the best fit for your research. If you are not sure, read the appropriate sections before making your selection.

[x] Life sciences [ ] Behavioural & social sciences [ ] Ecological, evolutionary & environmental sciences

For a reference copy of the document with all sections, see nature.com/documents/nr-reporting-summary-flat.pdf

# Life sciences study design

All studies must disclose on these points even when the disclosure is negative.

| | |
|---|---|
| Sample size | The number of animals per experimental group necessary to obtain a statistical significance (3Rs principle) was defined by determining:<br>- the statistical test to be used: T-test or ANOVA and post-hoc test (Tukey) for comparing two or more than two experimental groups, respectively.<br>- the expected biological effect and dispersion, this estimate was made based on bibliographic data and / or previous results obtained by the laboratory<br>- the power of the test was set at 0.8<br>- the 1st species risk was set at 0.05 |
| Data exclusions | No data were excluded from the analyses. |
| Replication | All attempts at replication were successful. |
| Randomization | Individuals were randomly allocated into experimental groups. |
| Blinding | The investigator was blinded to group allocation during data collection and analyses. |

# Reporting for specific materials, systems and methods

We require information from authors about some types of materials, experimental systems and methods used in many studies. Here, indicate whether each material, system or method listed is relevant to your study. If you are not sure if a list item applies to your research, read the appropriate section before selecting a response.

## Materials & experimental systems

| n/a | Involved in the study | |
|---|---|---|
| [ ] | [x] | Antibodies |
| [ ] | [x] | Eukaryotic cell lines |
| [x] | [ ] | Palaeontology and archaeology |
| [ ] | [x] | Animals and other organisms |
| [x] | [ ] | Human research participants |
| [x] | [ ] | Clinical data |
| [x] | [ ] | Dual use research of concern |

## Methods

| n/a | Involved in the study | |
|---|---|---|
| [x] | [ ] | ChIP-seq |
| [ ] | [x] | Flow cytometry |
| [x] | [ ] | MRI-based neuroimaging |

## Antibodies

| | |
|---|---|
| Antibodies used | Primary antibodies:<br>- Goat anti-human LDLR, AF2148, R&D Systems<br>- Goat anti-mouse LDLR, AF2255, R&D Systems<br>- Rabbit anti-human pan-Cytokeratin, ab9377, Abcam<br>- Alexa Fluor 594 goat anti-IgG, Fcγ fragment specific, 109-585-098, Jackson ImmunoResearch |
| Validation | Goat anti-human LDLR (AF2148, R&D Systems): its specificity against human LDLR has been validated by western-blot from LDLR knock-out (KO) versus wild-type LDLR BxPC3 cells.<br>Goat anti-mouse LDLR (AF2255, R&D Systems): its specificity against human LDLR has been validated by western-blot from LDLR knock-out (KO) versus LDLR PK4A cells.<br>Rabbit anti-human pan-Cytokeratin (ab9377, Abcam) reacts against human cytokeratin and validated in numerous publications, including Tu WJ et al. Targeting Nuclear LSD1 to Reprogram Cancer Cells and Reinvigorate Exhausted T Cells via a Novel LSD1-EOMES Switch. Front Immunol 11:1228 (2020).<br>Alexa Fluor 594 goat anti-IgG, Fcγ fragment specific (109-585-098, Jackson ImmunoResearch): its specificity has been validated in David et al. (2018) Identification and characterization of highly versatile peptide-vectors that bind non-competitively to the low-density lipoprotein receptor for in vivo targeting and delivery of small molecules and protein cargos. |

## Eukaryotic cell lines

Policy information about cell lines

| | |
|---|---|
| Cell line source(s) | Murine pancreatic adenocarcinoma cells (home-made) and PANC-1 (CRL-1469), MIA PaCa-2 (CRL-1420) and BxPC-3 (CRL-1687) come from American Tissue Cell Culture (ATCC, USA). |
| Authentication | The murine pancreatic adenocarcinoma cells, namely PK4A, have been authenticated by their expression of characteristic epithelial markers (cytokeratin, CK19, E cadherin) and epthelial to mesenchymal transition markers (N cadherin, Vimentin) and previously published in Guillaumond et al, 2013 Proc Natl Acad Sci U S A. 2013 Mar 5;110(10):3919-24.<br><br>The BxPC3-, MIA PaCa-2 and PANC-1 were authenticated using short tandem repeat analysis, as described in 2012 in ANSI Standard by the ATCC standards development organization (SDO) and in Capes-Davis et al. (Int. J. Cancer, 2012). The submitted profile of each cell line is an exact match for the following ATCC human cell line in the ATCC STR database. Detailed STR profile report are available upon request. |
| Mycoplasma contamination | All cell lines used were tested negative for mycoplasma before experiments (information stated in Methods section. |
| Commonly misidentified lines<br>(See ICLAC register) | *Name any commonly misidentified cell lines used in the study and provide a rationale for their use.* |

## Animals and other organisms

Policy information about studies involving animals; ARRIVE guidelines recommended for reporting animal research

| | |
|---|---|
| Laboratory animals | - KIC mice: LSL-KRasG12D; Ink4a/Arffl/fl; Pdx1-Cre (9-weeks old), and their age-matched control mice (KI: LSL-KRasG12D; Ink4a/Arffl/fl), males and females.<br>- KI mice : LSL-KRasG12D; Ink4a/Arffl/fl, 7-weeks old, males and females.<br>- 5 week-old female athymic mice (Hsd:Athymic Nude-Foxn1nu, Envigo, France). |
| Wild animals | This study does not involve wild animals. |
| Field-collected samples | All mice were housed under regulated temperature 22 ± 2°C and 12h-Light/12h-dark cycle. They received water and food ad libitum. Each cage contained 5 mice.<br>All experiments have been stopped when the tumor volume reaches 300 mm3. |
| Ethics oversight | Mice breeding and all experimental procedures were approved by the local ethics committee for animal experimentation (CEEA14, Marseille, France) (N°01527-02, N°9749-2017042710417337 and N°24209-2020021719581546). |

Note that full information on the approval of the study protocol must also be provided in the manuscript.

## Flow Cytometry

### Plots

Confirm that:

- [x] The axis labels state the marker and fluorochrome used (e.g. CD4-FITC).
- [x] The axis scales are clearly visible. Include numbers along axes only for bottom left plot of group (a 'group' is an analysis of identical markers).
- [ ] All plots are contour plots with outliers or pseudocolor plots.
- [ ] A numerical value for number of cells or percentage (with statistics) is provided.

### Methodology

| | |
|---|---|
| Sample preparation | Adherent pancreatic cancer cells were incubated with the fluorescent conjugate in presence or not of DiI-LDL. Then, half of the cells were treated with an acid stripping solution (0.15 M NaCl, 0.2 M Glycine, pH 3) to remove the cell-surface receptors. Finally, all cells treated or not with an acid stripping solution were detached with accutase cell detachment solution, centrifuged and rinced before being fixed in 2% PFA and 5 mM EDTA. |
| Instrument | Samples were acquired using Attune NxT flow cytometer (ThermoFisher) |
| Software | FACS analysis were done using Attune NxT Software version 3.1 (ThermoFisher). |
| Cell population abundance | The cell population, directly derived from the different cell culture experiments, was acquired and analysed by flow cytometry. |
| Gating strategy | First a FSC/SSC gate was performed to select the interest cell population based on their size and granularity, the cell debris and dead cells (in the bottom left corner) were excluded (P1 population). Then, a doublet exclusion (FSC-H/FSC-A, SSC-H/SSC-A) in the subpopulation P1 was performed to end up with high purity samples. Finally, to accurately identified the positive cancer cells (stained with fluorochorme of interest), FACS analysis was repeated with unstained cells to manually set a |

positivity treshold to partition events into fluorescent-positive and fluorescent-negative population.

☒ Tick this box to confirm that a figure exemplifying the gating strategy is provided in the Supplementary Information.
